# Supplementary material for: Navigating the Chemical Space of ENR Inhibitors: A Comprehensive Analysis
Source: Antibiotics (Basel). 2024 Mar 11;13(3):252. doi: 10.3390/antibiotics13030252 (PMC10967325; doi:10.3390/antibiotics13030252)
Supplement: Supplementary file 1 [file antibiotics-13-00252-s001.zip › antibiotics-2877387-supplementary.pdf]

Navigating the Chemical Space of ENR Inhibitors: A Comprehensive Analysis

Vid Kuralt and Rok Frlan*

Department of Pharmaceutical Chemistry, Faculty of Pharmacy, University of Ljubljana, 1000 Ljubljana, Slovenia; kuraltvid@gmail.com
* Correspondence: rok.frlan@ffa.uni-lj.si; Tel.: +386-1-4769-674

Contents

Summary statistics for data collection 2
Calculation of molecular descriptors 3
Statistics 4
Normal distribution testing 4
Distribution of features 5
Mann–Whitney U tests 15
Statistical values of selected features 19
Temporal Progression of Inhibitors' Physicochemical Features for Each Enzyme 22
Lipinski violations 24
Pan-assay interference compounds (PAINS) and BRENK compounds 24
Molecular similarity 26
Molecular complexity 27
Analysis of clusters 28
Matched molecular pairs (MMP) 28
SHAP analysis 36

Summary statistics for data collection

Table S1. Summary statistics of enzymes included in the study, categorized by source.

Enzyme	Source	Activity	Count	Percent (%)[a]
FabI	BindingDB	Active	81	5.7
FabI	BindingDB	Inactive	30	2.1
FabI	ChEMBL	Active	185	13.1
FabI	ChEMBL	Inactive	120	8.5
FabK	BindingDB	Active	6	0.4
FabK	ChEMBL	Active	18	1.3
FabK	ChEMBL	Inactive	16	1.1
FabV	BindingDB	Active	3	0.2
FabV	BindingDB	Inactive	7	0.5
InhA	BindingDB	Active	5	0.4
InhA	ChEMBL	Active	26	1.8
InhA	ChEMBL	Inactive	40	2.8
InhA	Literature	Active	231	16.4
InhA	Literature	Inactive	598	42.4
InhA	OurDB	Active	2	0.1
InhA	OurDB	Inactive	44	3.1
FabI	BindingDB	Active	81	5.7

[a]The ratio of individual entry per number of all compounds combined. Percents may not add up to one hundred due to rounding

Table S2. Summary statistics of active enzymes by source.

Enzyme	Source	Activity	Count	Percent (%)[a]
FabI	BindingDB	Active	81	14.5
FabI	ChEMBL	Active	185	33.2
FabK	BindingDB	Active	6	1.1
FabK	ChEMBL	Active	18	3.2
FabV	BindingDB	Active	3	0.5
InhA	BindingDB	Active	5	0.9
InhA	ChEMBL	Active	26	4.7
InhA	Literature	Active	231	41.5
InhA	OurDB	Active	2	0.4

[a]The ratio of each entry to the total number of active compounds. Percents may not add up to one hundred due to rounding

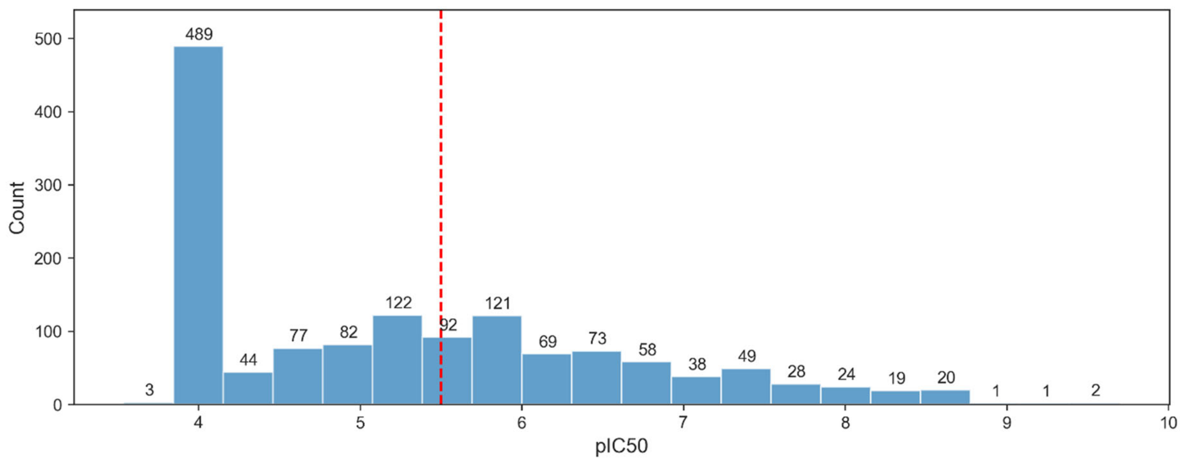

Figure S1. Histogram of pIC_{50} values for all enzymes with a dashed threshold line at $pIC_{50} = 5.5$ on the X-axis. The total number of compounds is 1413.

Calculation of molecular descriptors

RDKit descriptors were generated using the following features:

- Enhanced/hybrid logP (SlogP)
- Molecular refractivity (SMR)
- Labute's Approximate Surface Area (LabuteASA)
- Total polar surface area (TPSA)
- Average molar weight (AMW)
- Exact molar weight (ExactMW)
- Number of rotatable bonds (NumRotatableBonds)
- Number of hydrogen bond donors (NumHBD)
- Number of hydrogen bond acceptors (NumHBA)
- Number of amide bonds (NumAmideBonds)
- Number of hetero atoms (NumHeteroAtoms)
- Number of atoms (NumAtoms)
- Number of stereocenters (NumStereocenters)
- Number of unspecified stereocenters (NumUnspecifiedStereocenters)
- Number of rings (NumRings)
- Number of aromatic rings (NumAromaticRings)
- Number of saturated rings (NumSaturatedRings)
- Number of aliphatic rings (NumAliphaticRings)
- Number of aromatic heterocycles (NumAromaticHeterocycles)
- Number of saturated heterocycles (NumSaturatedHeterocycles)
- Number of aliphatic heterocycles (NumAliphaticHeterocycles)
- Number of aromatic carbocycles (NumAromaticCarbocycles)
- Number of saturated carbocycles (NumSaturatedCarbocycles)
- Number of aliphatic carbocycles (NumAliphaticCarbocycles)
- Fraction of carbon atoms that are sp3 hybridized (FractionCSP3)
- HallKier Alpha (represents the connectivity index of a given molecule)

In total, 29 features were used.

Statistics

Normal distribution testing

Table S3. Results of normality testing using the Kolmogorov–Smirnov test. The table presents *p*-values, where '_a' denotes active and '_i' denotes inactive.

Features	InhA_a	InhA_i	FabI_a	FabI_i	FabK_i	Dataset_a	Dataset_i
BertzCT	0.047	0	0.105	0.07	0.789	0	0
fragCpx	0.001	0	0.008	0.002	0.346	0	0
FilterItLogS	0.063	0.001	0.244	0.026	0.463	0	0
apol	0	0	0.001	0	0.51	0.002	0
FMF	0	0	0	0	0	0	0
RotBFrac	0	0	0	0	0.941	0	0
RotBtFrac	0	0	0.005	0	0.773	0	0
SlogP	0.006	0	0.004	0.483	0.275	0.022	0.004
TPSA	0	0	0.239	0	0.519	0	0
ExactMW	0	0	0.017	0	0.347	0	0

Distribution of features

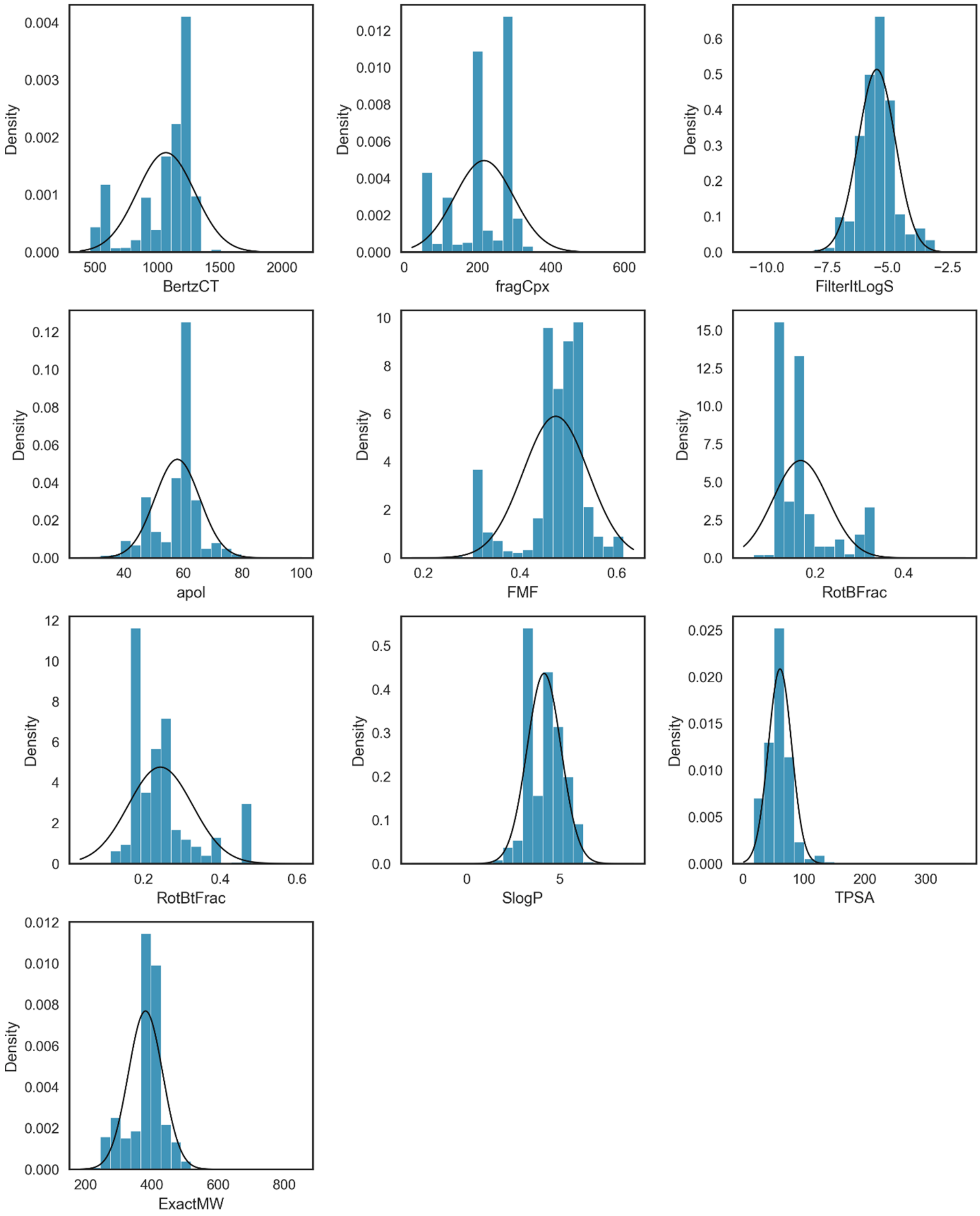

Figure S2. Feature distribution in the entire dataset of active compounds. Black graphs represent calculated normal distribution.

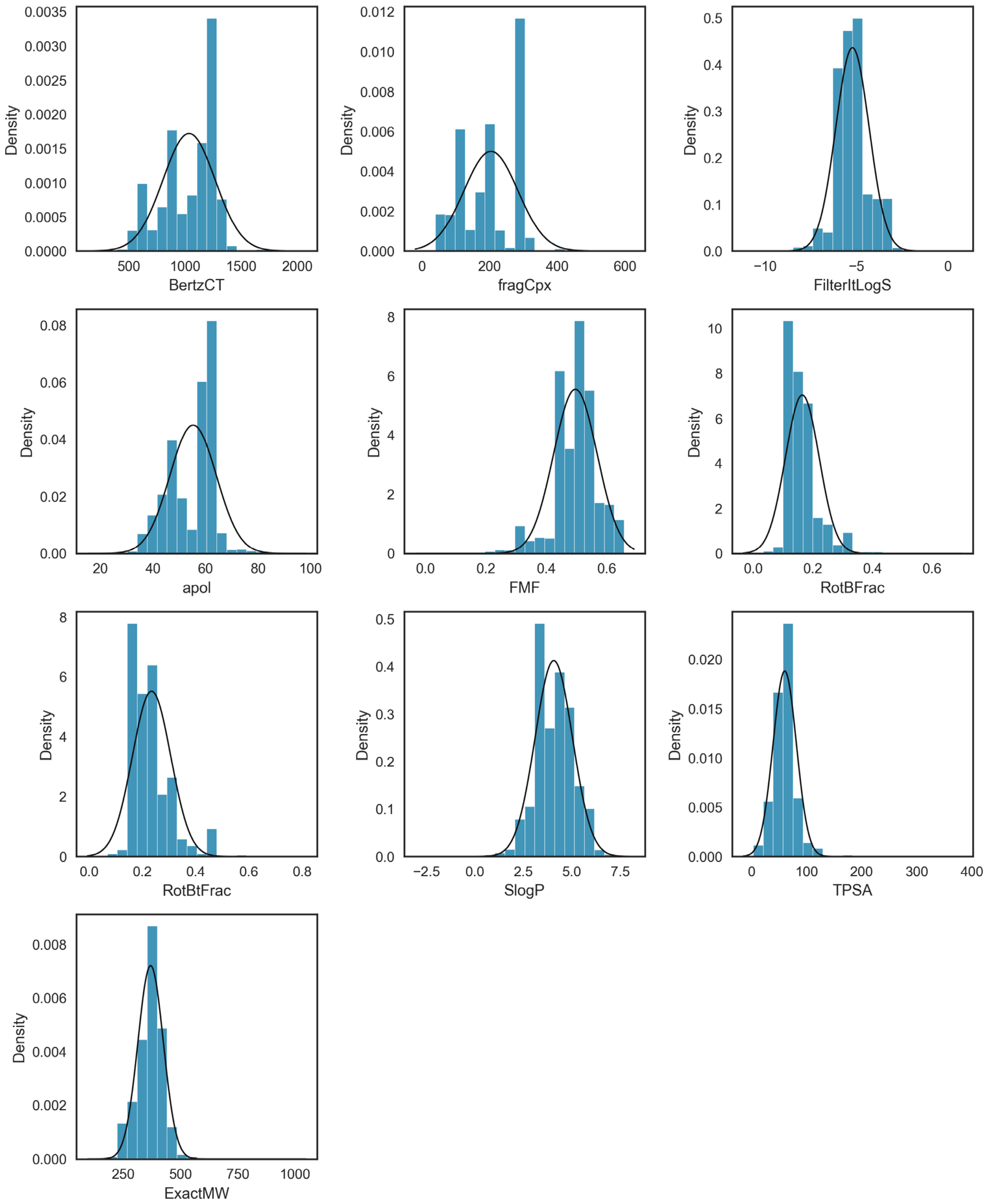

Figure S3. Feature distribution in the dataset of inactive compounds. Black graphs represent calculated normal distribution.

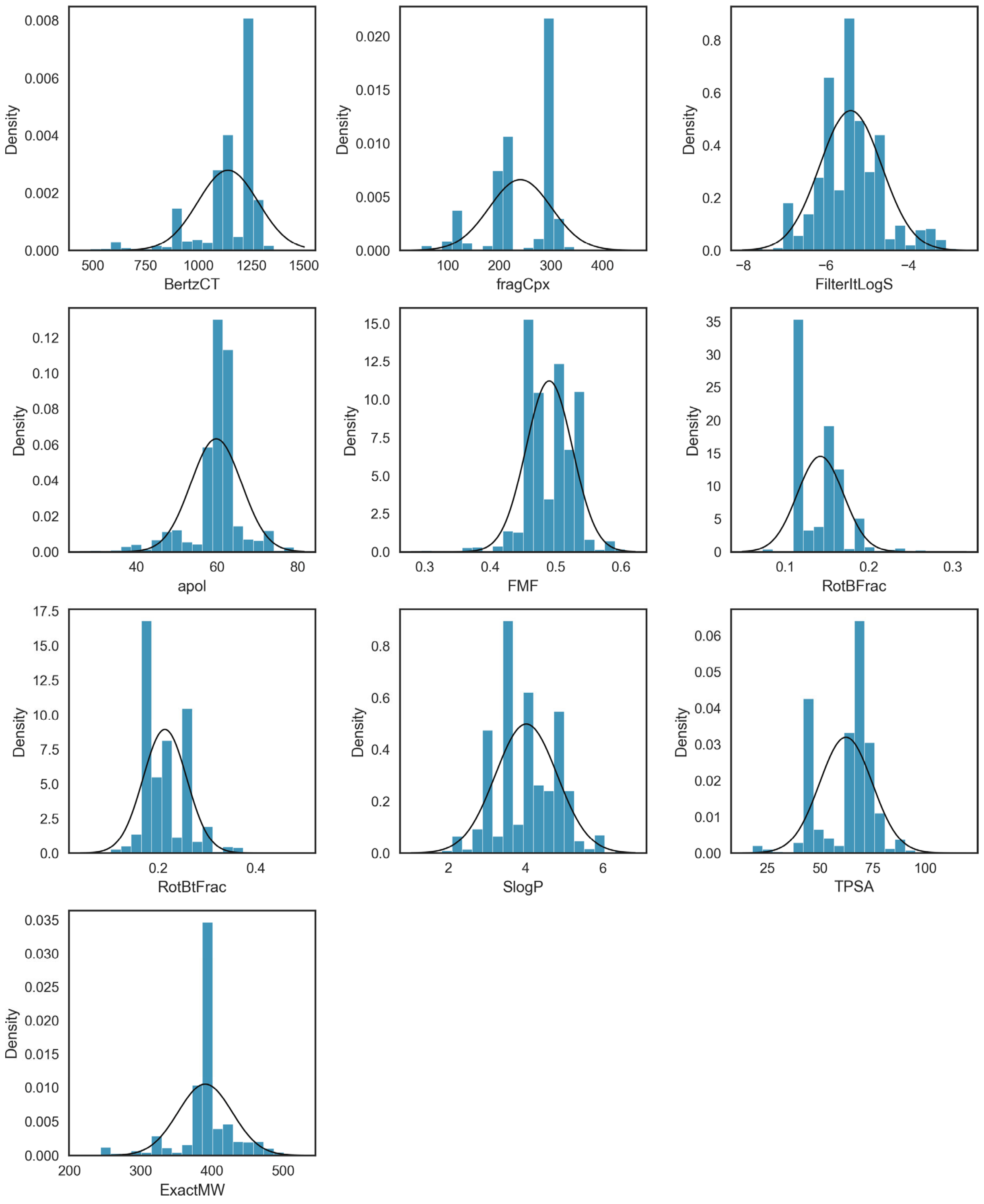

Figure S4. Feature distribution in the FabI dataset of active compounds. Black graphs represent calculated normal distribution.

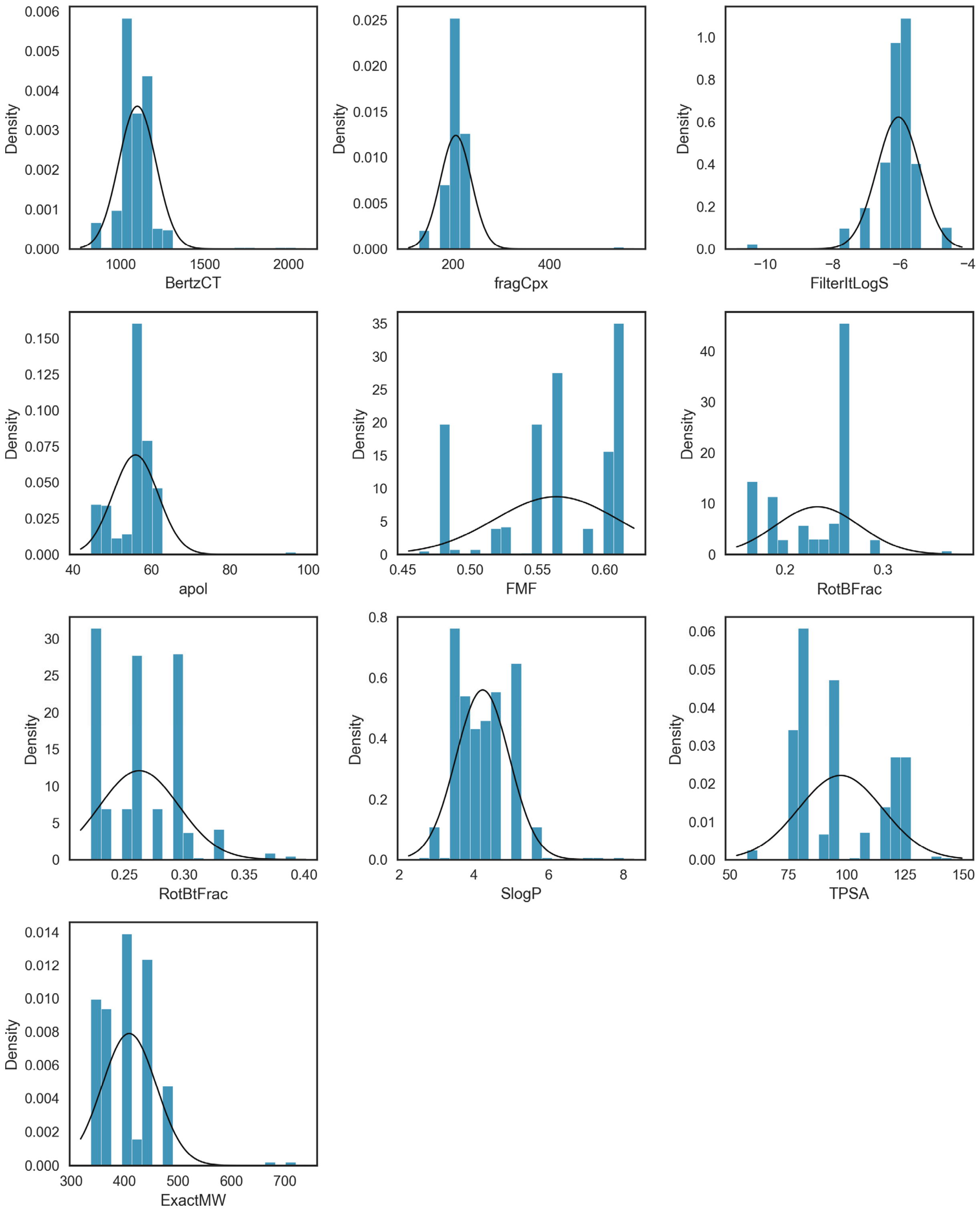

Figure S5. Feature distribution in the FabK dataset of active compounds. Black graphs represent calculated normal distribution.

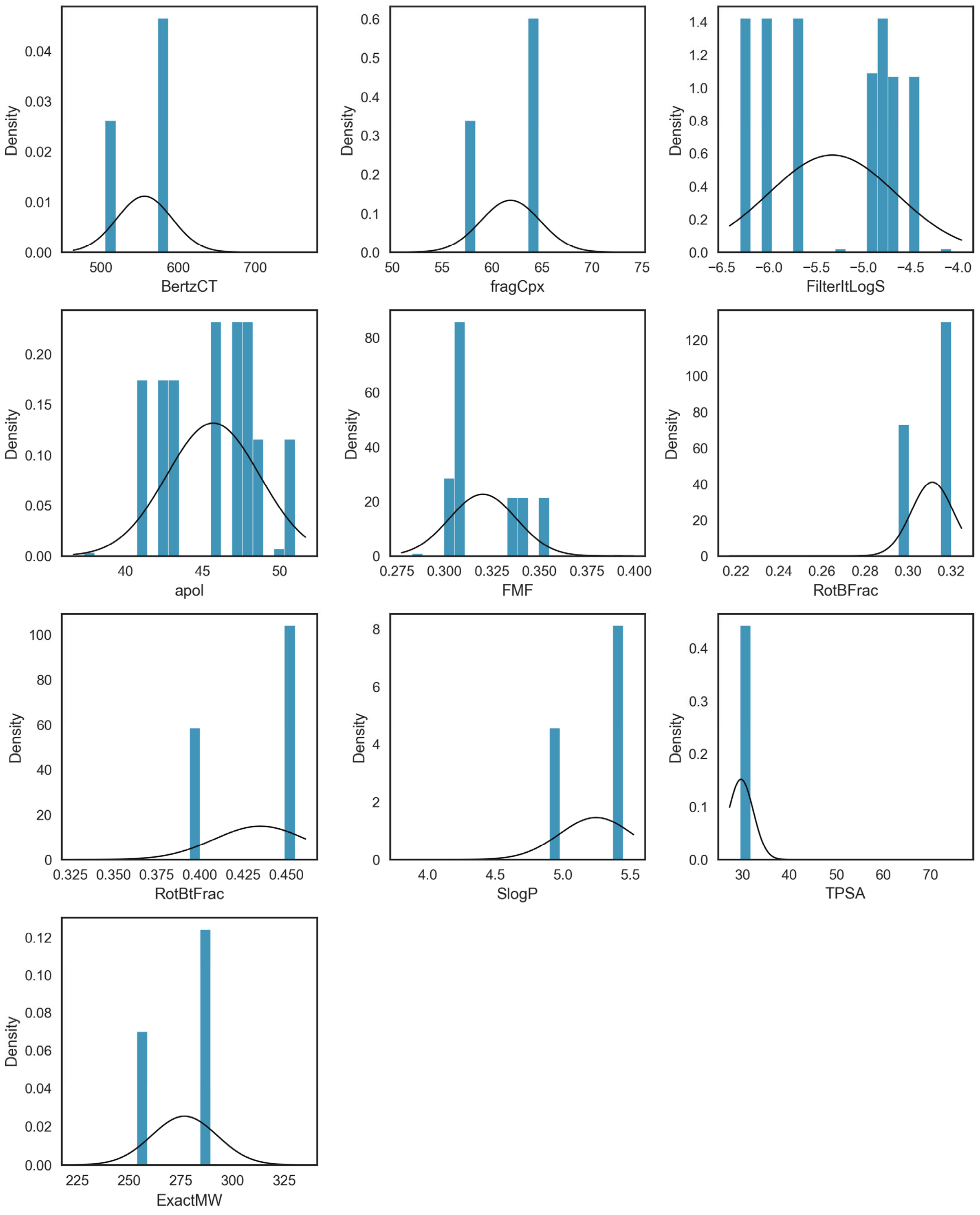

Figure S6. Feature distribution in the FabV dataset of active compounds. Black graphs represent calculated normal distribution.

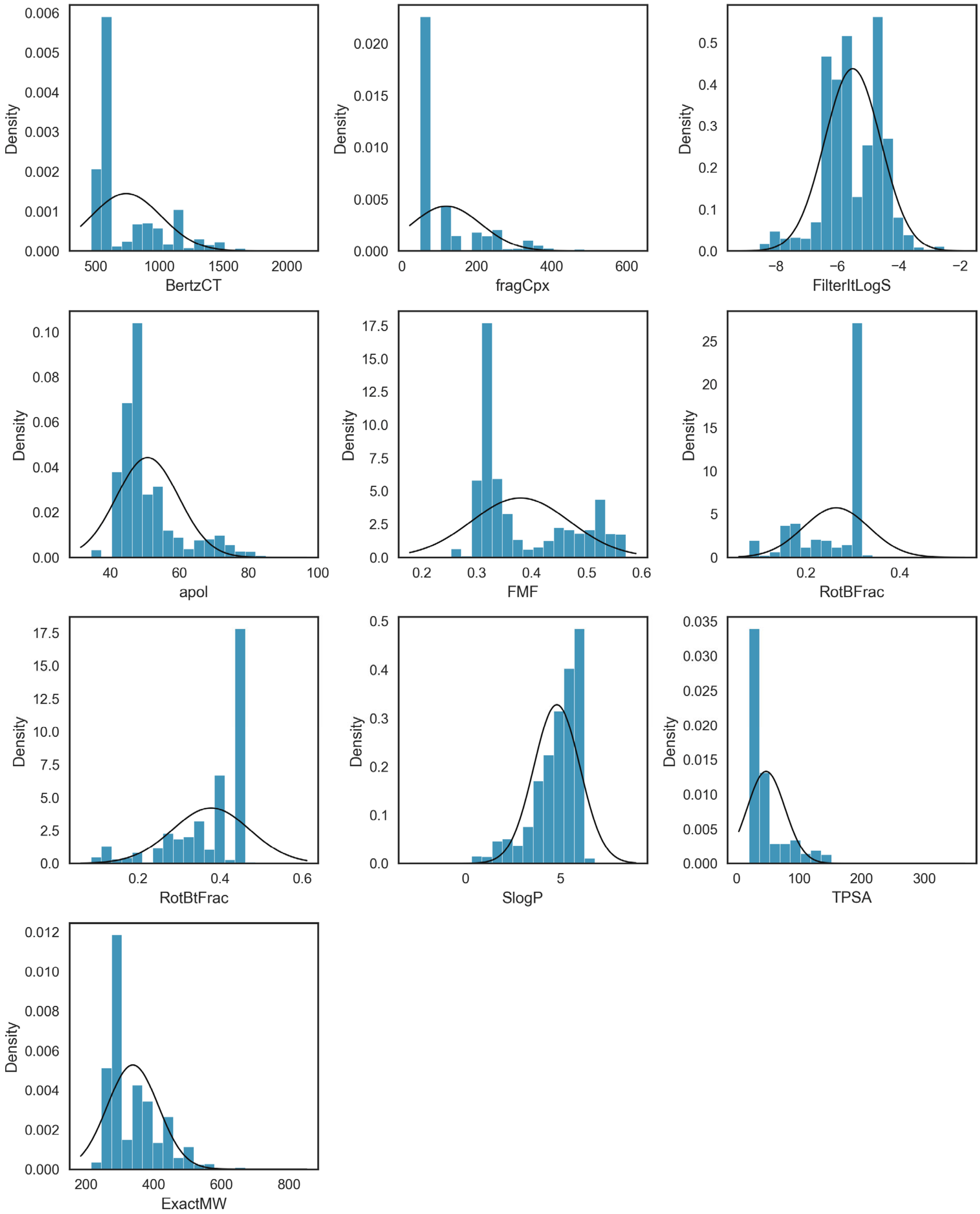

Figure S7. Feature distribution in the InhA dataset of active compounds. Black graphs represent calculated normal distribution.

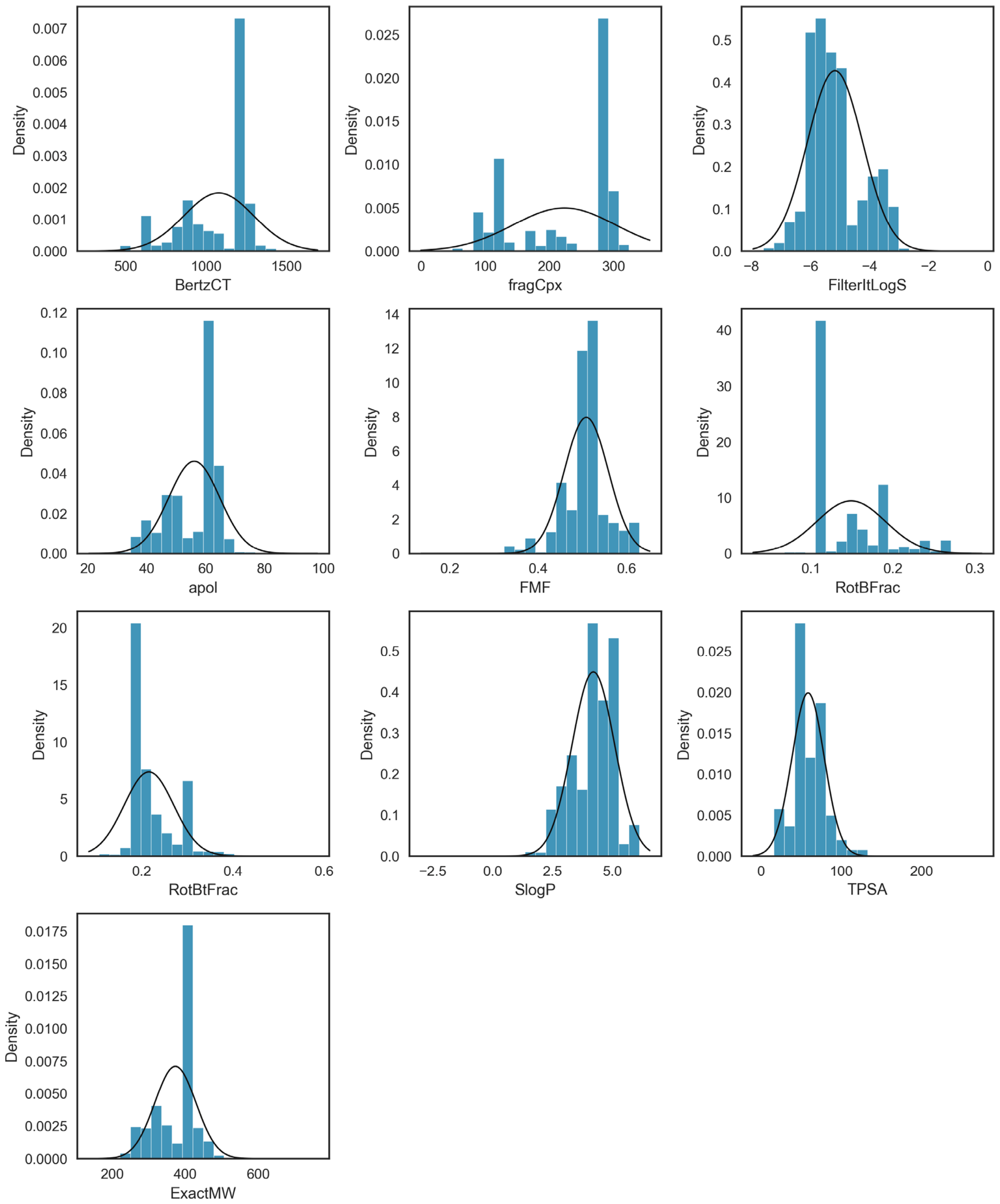

Figure S8. Feature distribution in the FabI dataset of inactive compounds. Black graphs represent calculated normal distribution.

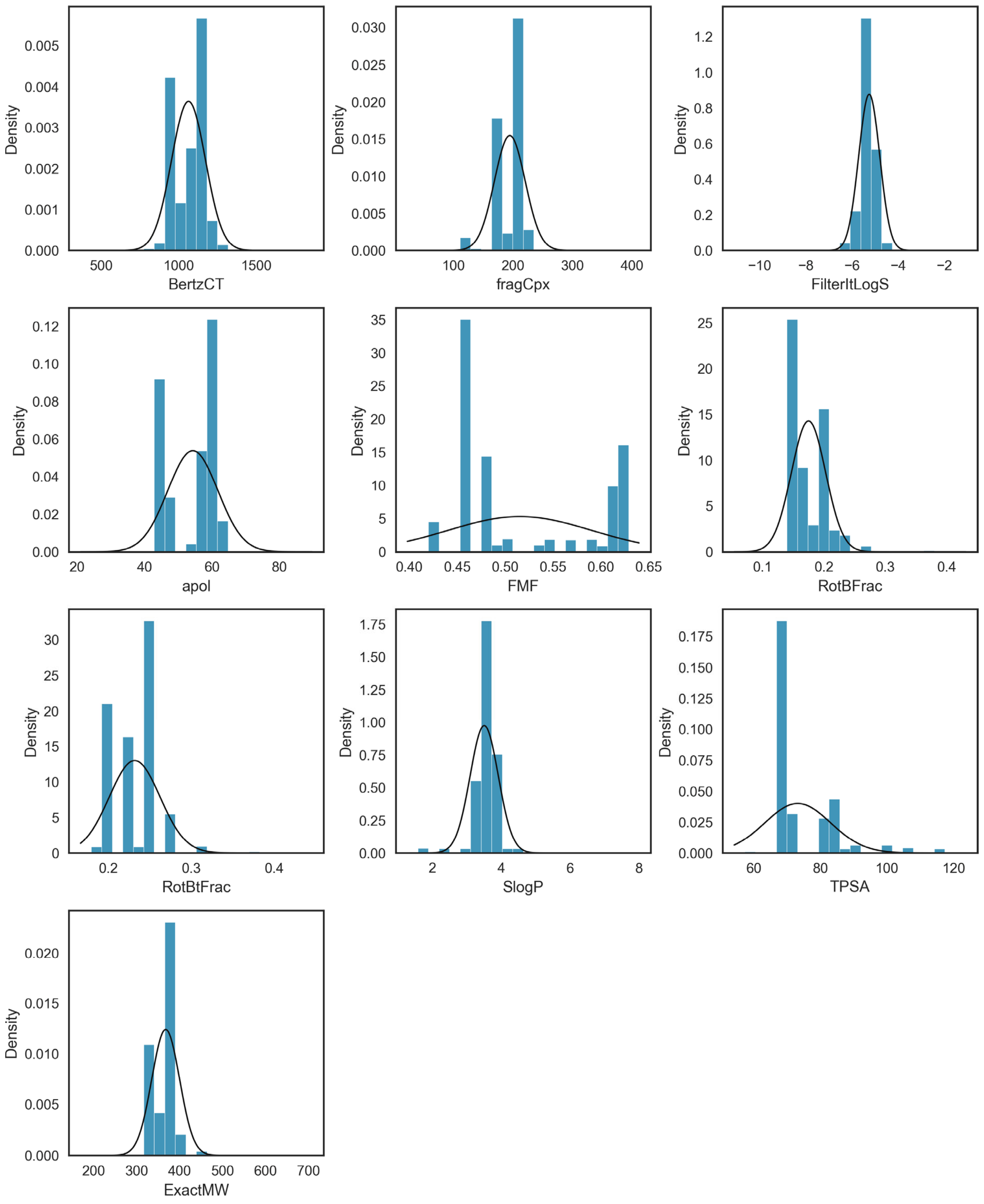

Figure S9. Feature distribution in the FabK dataset of inactive compounds. Black graphs represent calculated normal distribution.

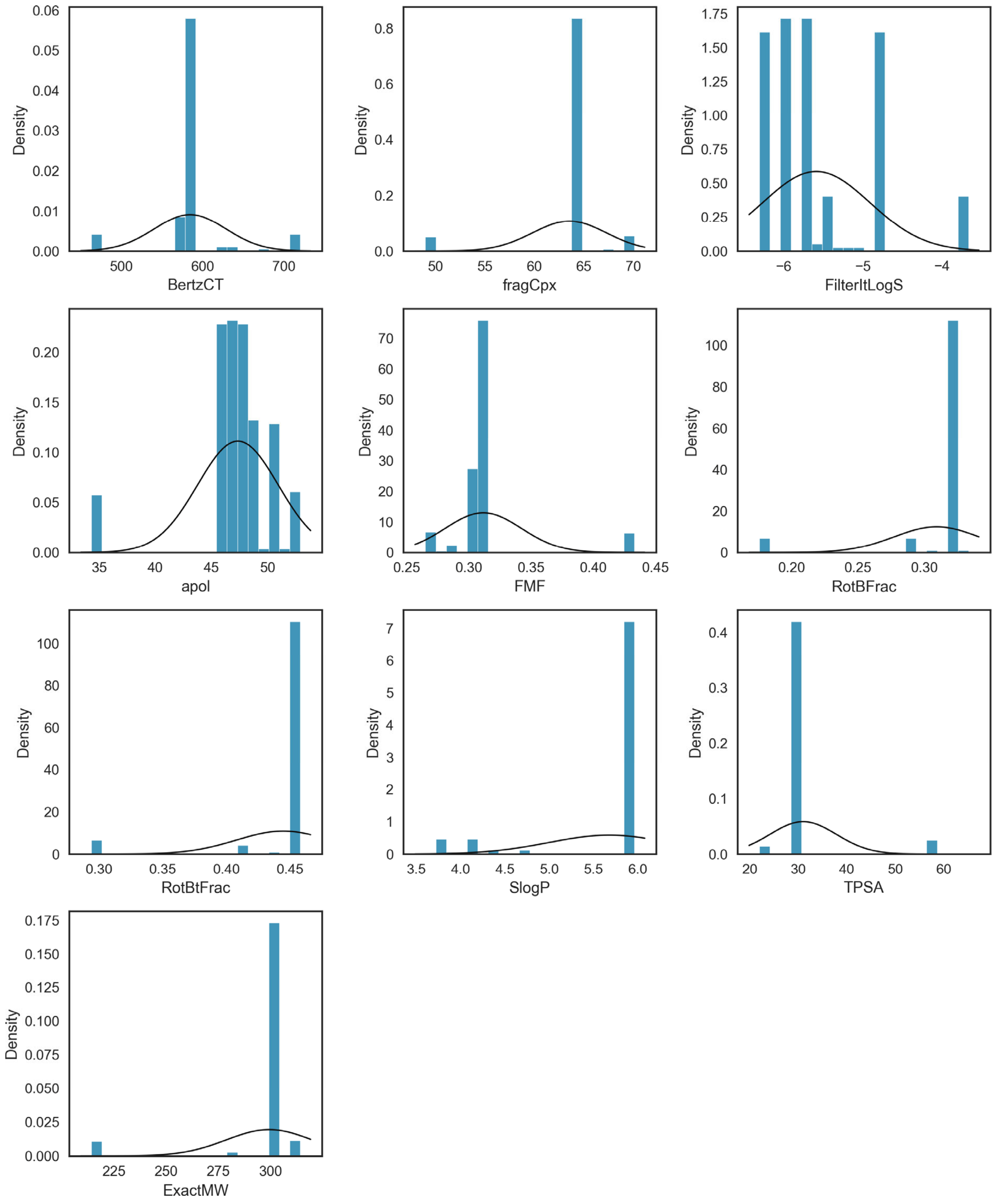

Figure S10. Feature distribution in the FabV dataset of inactive compounds. Black graphs represent calculated normal distribution.

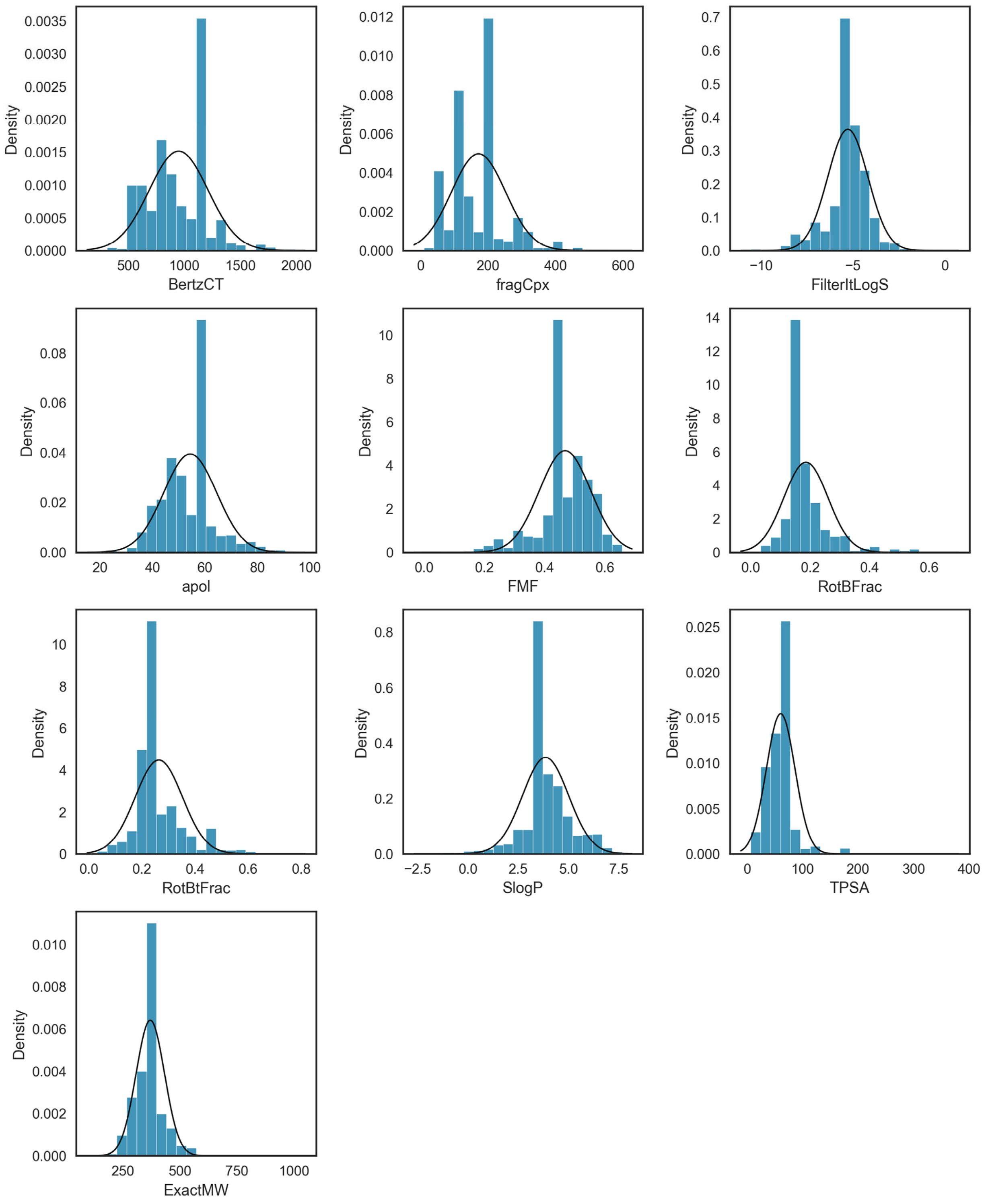

Figure S11. Feature distribution in the InhA dataset of inactive compounds. Black graphs represent calculated normal distribution.

Mann-Whitney U tests

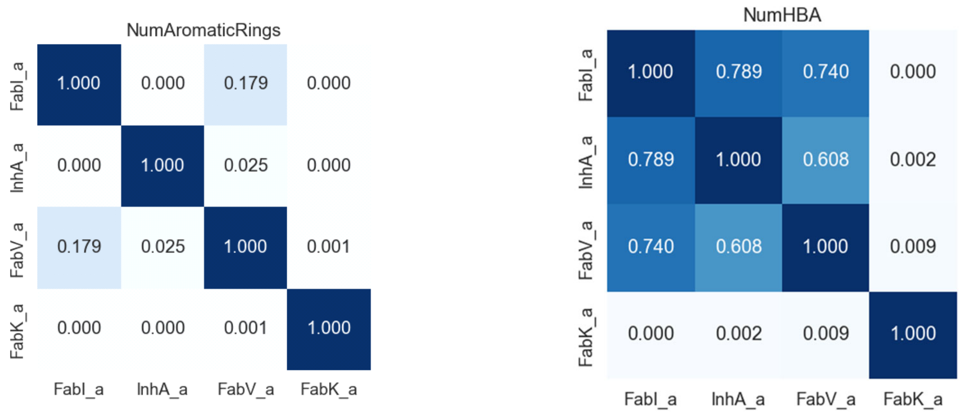

Figure S12. Heatmap of Mann–Whitney U test *p*-values: Comparisons of the number of aromatic rings(NumAromaticRings) and the number of hydrogen bond accepting groups (NumHBA) across all four enzyme datasets of active compounds.

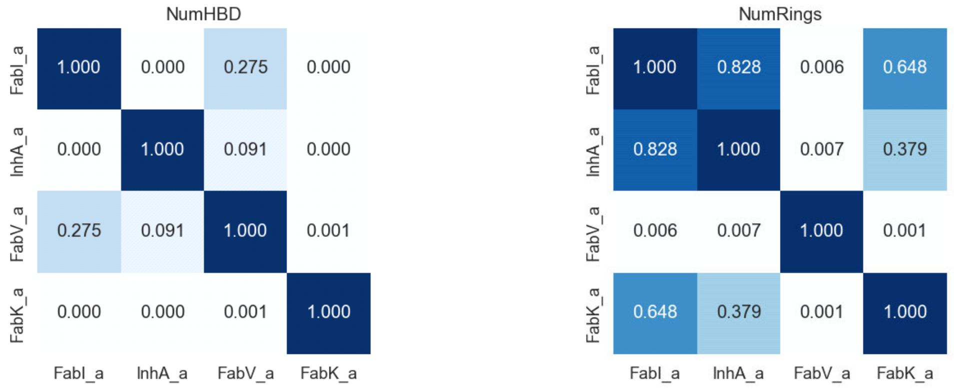

Figure S13. Heatmap of Mann–Whitney U test *p*-values: Comparisons of the number of aromatic rings(NumAromaticRings) and the hydrogen bond accepting groups (NumHBA) across all four enzyme datasets of active compounds.

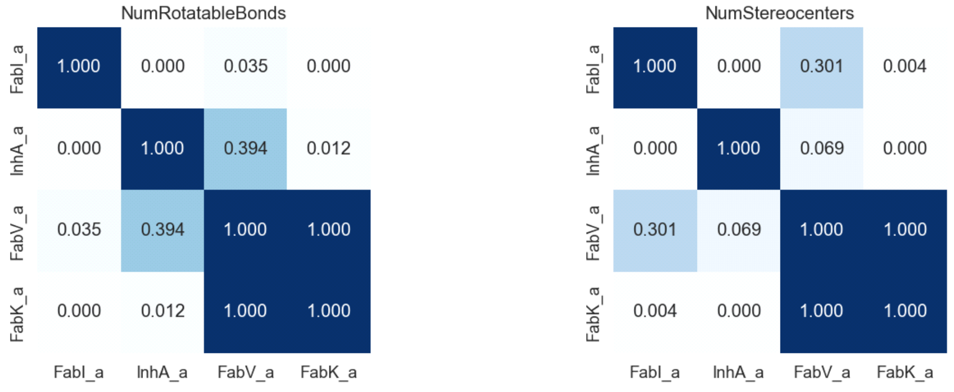

Figure S14. Heatmap of Mann–Whitney U test *p*-values: Comparisons of the number of rotatable bonds (NumRotatableBonds) and the number of stereocenters (NumStereocenters) across all four enzyme datasets of active compounds.

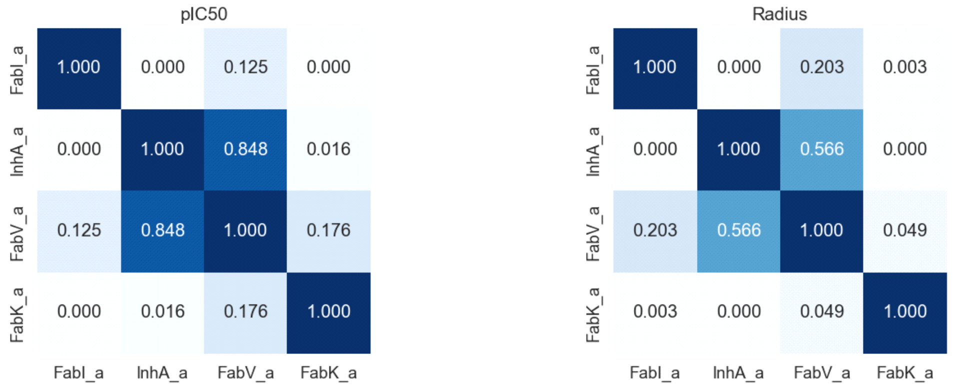

Figure S15. Heatmap of Mann–Whitney U test *p*-values: Comparisons of the pIC_{50} values and radius across all four enzyme datasets of active compounds.

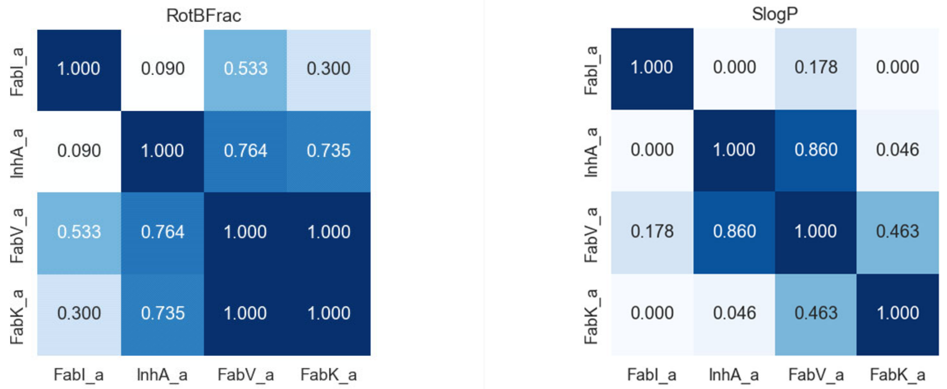

Figure S16. Heatmap of Mann–Whitney U test *p*-values: Comparisons of the fraction of rotatable bonds, excluding terminal bonds (RotBFrac) and SlogP radius across all four enzyme datasets of active compounds.

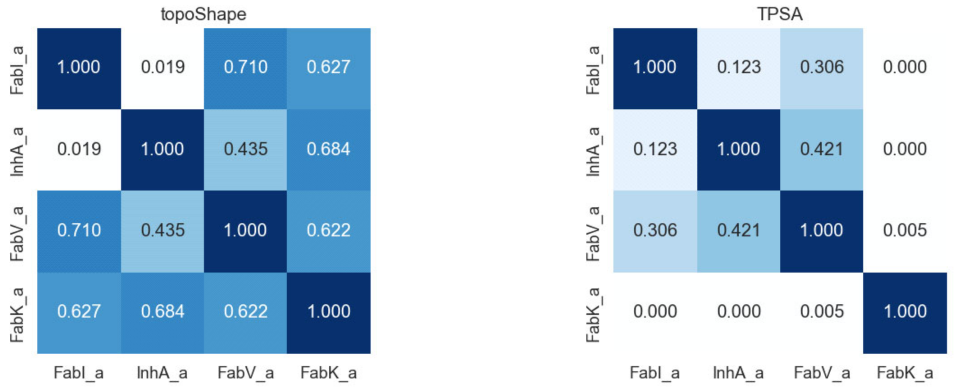

Figure S17. Heatmap of Mann–Whitney U test *p*-values: Comparisons of the petitjean topological shape index (topoShape) and topological polar surface area(TPSA) across all four enzyme datasets of active compounds.

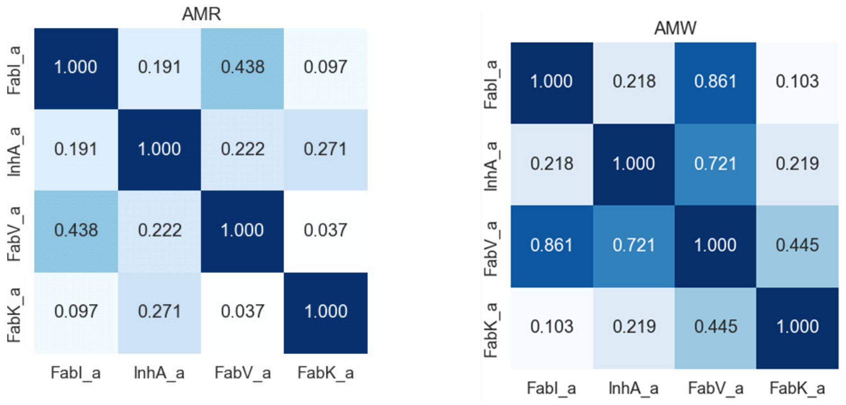

Figure S18. Heatmap of Mann–Whitney U test *p*-values: Comparisons of the molar refractivity(AMR) and average molecular weight (AMW) across all four enzyme datasets of active compounds.

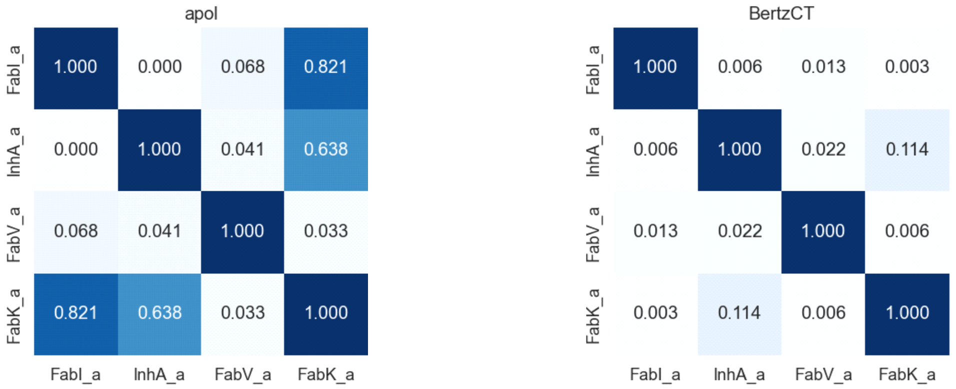

Figure S19. Heatmap of Mann–Whitney U test *p*-values: Comparisons of the sum of the atomic polarizabilities (apol) and BertzCT across all four enzyme datasets of active compounds.

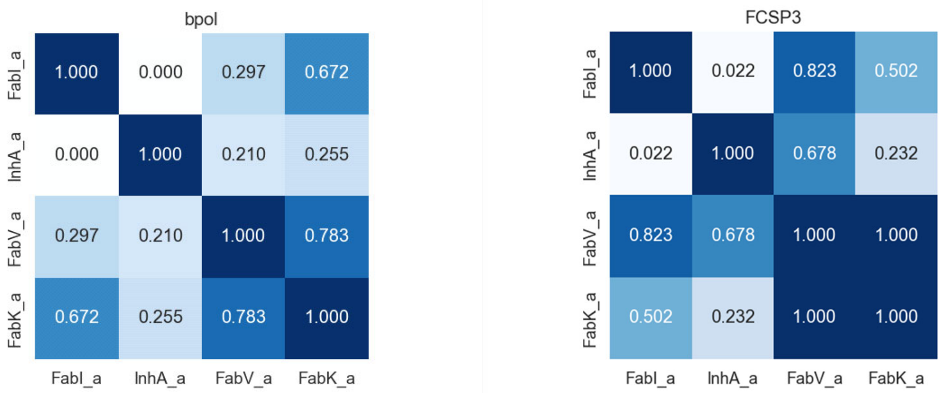

Figure S20. Heatmap of Mann–Whitney U test *p*-values: Comparisons of the BPolDescriptor (bpol) and fraction of carbons that are sp3 (FCSP3) across all four enzyme datasets of active compounds.

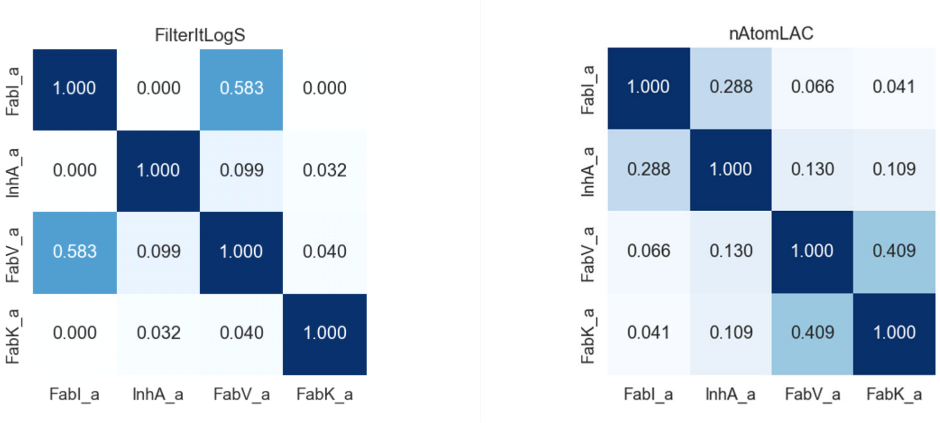

Figure S21. Heatmap of Mann–Whitney U test *p*-values: Comparisons of the FilterItLogS and longest aliphatic chain (nAtomLAC) across all four enzyme datasets of active compounds.

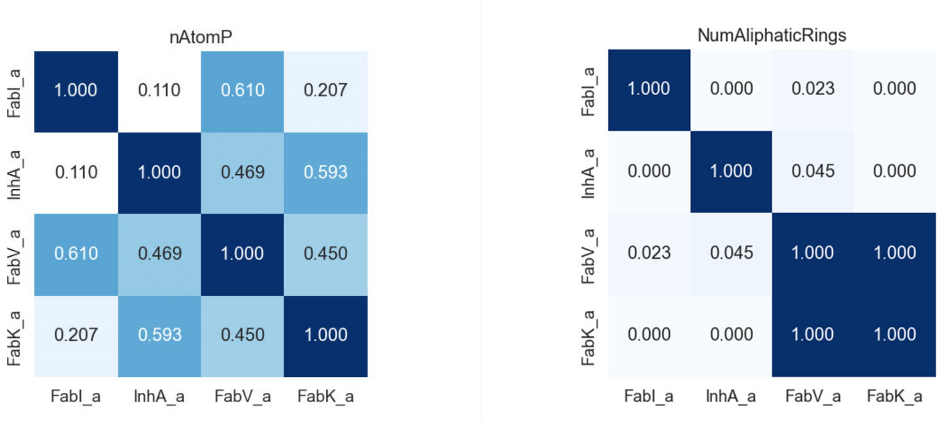

Figure S22. Heatmap of Mann–Whitney U test *p*-values: Comparisons of the number of atoms in the largest pi system (nAtomP) and the number of aliphatic rings (NumAliphaticRings) across all four enzyme datasets of active compounds.

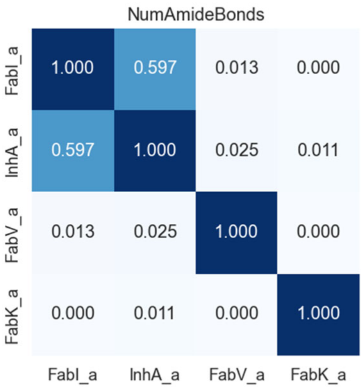

Figure S23. Heatmap of Mann–Whitney U test *p*-values: Comparisons of the number of amide bonds (NumAmideBonds) across all four enzyme datasets of active compounds.

Statistical values of selected features

Table S4. Median values for calculated selected features for all four datasets.

Enzyme	FabI		FabK		FabV		InhA	
**Activity**	**Active**	**Inactive**	**Active**	**Inactive**	**Active**	**Inactive**	**Active**	**Inactive**
AMR	48	45	54.5	42.5	41	34	52	41
AMW	7	8	7	8.5	7	8	7	8
BertzCT	977	861.5	1072	1090	660	627	1004	868.5
FCSP3	0	0	0	0	0	0	0	0
FilterItLogS	−4.941	−4.534	−6.098	−5.446	−4.885	−5.55	−5.654	−5.202
NumAliphaticRings	1	1	0	0	0	0	1	1
NumAmideBonds	2	1	2	2	0	0	1	1
NumAromaticRings	2	3	4	4	2	2	3	3
NumHBA	4	4	6	5.5	4	3	4	4
NumHBD	1	1	3	3	1	0	1	1
NumRings	4	3	4	4	2	2	4	3
NumRotatableBonds	4	4	6	4	7	7	5	4
NumStereocenters	0	0	0	0	0	0	1	0
Radius	8	7	9	8	8	7	8	7
RotBFrac	0.224	0.183	0.236	0.191	0.238	0.15	0.233	0.161
SlogP	3.526	3.305	4.613	3.548	4.137	4.019	4.232	3.905
TPSA	66.25	65.5	100.15	102.9	57.3	31.2	69.6	57.4
apol	57.8	48.3	56.8	48.3	49.9	50.3	60.8	52.2
bpol	28	24	27	26.5	28	28	30	25
nAtomLAC	2	0	3	0	4	0	2	0
nAtomP	12	14	11	9.5	14	18	12	12
pIC50	6.063	5	7.45	4.798	6.699	4.377	6.699	4
topoShape	0.9	0.92	0.976	0.958	0.889	1	1	0.889

Table S5. Mean values for calculated selected features for all four datasets.

Enzyme	FabI		FabK		FabV		InhA	
Activity	Active	Inactive	Active	Inactive	Active	Inactive	Active	Inactive
AMR	48.944	50.167	59	44.312	40.333	34.286	50.723	42.975
AMW	7.485	8.293	7.125	8.75	7.667	8.143	7.386	8.255
BertzCT	965.594	866.253	1188.062	1192.562	629.667	641.429	1049.451	914.989
FCSP3	0.019	0.033	0	0	0	0	0.057	0.174
FilterItLogS	−5.045	−4.476	−6.611	−6.455	−4.731	−5.475	−5.71	−5.301
NumAliphaticRings	1.429	0.887	0	0.062	0	0	0.966	0.773
NumAmideBonds	1.323	0.96	1.958	1.688	0	0	1.417	0.896
NumAromaticRings	2.515	2.507	4.167	3.875	2	2	2.977	2.705
NumHBA	4.064	4.247	6.292	5.188	3.667	2.714	4.739	4.248
NumHBD	1.064	1.267	2.917	2.688	0.667	0.286	1.439	1.035
NumRings	3.944	3.393	4.167	3.938	2	2	3.943	3.478
NumRotatableBonds	4.019	3.82	6.667	5.125	6.333	7.143	5.447	5.075
NumStereocenters	0.459	0.5	0	0	0	0	0.958	0.535
Radius	8.15	7.287	9.5	8.688	7.333	7.286	7.837	7.468
RotBFrac	0.244	0.186	0.272	0.195	0.266	0.155	0.255	0.167
SlogP	3.608	3.375	4.703	4.307	4.076	4.153	3.951	3.887
TPSA	65.345	68.208	107.888	95.894	53.733	36.9	75.143	64.088
apol	55.522	50.031	60.679	56.725	45.767	49.929	61.033	53.854
bpol	27.65	24.38	29.208	26.438	24.667	27.286	30.803	26.336
nAtomLAC	2.624	1.267	5.125	0.562	4.667	0.857	3.144	1.095
nAtomP	12.914	13.167	11.75	11.312	14.333	15.143	12.322	13.087
pIC50	6.364	4.812	7.364	4.871	6.799	4.405	6.88	4.3
topoShape	0.93	0.933	0.936	0.941	0.915	0.932	0.941	0.926

Table S6. Standard deviation values for calculated selected features for all four datasets.

Enzyme	FabI		FabK		FabV		InhA	
Activity	Active	Inactive	Active	Inactive	Active	Inactive	Active	Inactive
Feature								
AMR	18.56	18.367	28.932	9.214	8.021	10.275	17.031	16.094
AMW	0.908	1.272	1.035	1.528	1.155	0.378	0.912	1.191
BertzCT	201.388	215.12	336.225	435.232	139.005	45.025	320.4	291.414
FCSP3	0.136	0.18	0	0	0	0	0.232	0.38
FilterItLogS	0.907	1.19	1.907	3.019	0.596	0.282	1.135	1.433
NumAliphaticRings	1.077	0.959	0	0.25	0	0	0.869	0.959
NumAmideBonds	0.86	0.933	0.359	0.704	0	0	1.147	0.805
NumAromaticRings	0.733	0.817	0.702	1.025	0	0	0.841	1.081
NumHBA	0.963	1.81	1.301	1.109	1.528	0.756	2.687	2.271
NumHBD	0.632	1.496	0.654	0.602	0.577	0.488	0.904	1.076
NumRings	0.998	0.926	0.702	0.998	0	0	1.224	1.219
NumRotatableBonds	1.307	1.182	2.036	2.754	2.082	0.378	2.014	3.107
NumStereocenters	0.796	0.809	0	0	0	0	1.2	1.312
Radius	1.25	1.435	1.978	2.915	1.155	0.488	1.15	1.527
AMWRotBFrac	0.099	0.055	0.126	0.032	0.094	0.02	0.098	0.052

SlogP	0.972	1.188	1.438	2.124	0.171	0.396	1.484	1.468
TPSA	18.093	34.533	19.522	15.113	22.661	17.03	40.609	36.843
apol	9.036	9.961	14.809	20.154	7.333	1.547	12.279	12.21
bpol	5.363	6.109	7.384	8.733	5.774	1.254	7.559	7.774
nAtomLAC	3.18	1.721	5.392	1.031	1.155	1.069	3.881	1.439
nAtomP	4.396	4.071	3.848	4.6	5.508	5.273	4.127	4.439
pIC50	0.753	0.528	0.962	0.371	0.174	0.262	0.894	0.479
topoShape	0.07	0.071	0.072	0.062	0.075	0.085	0.07	0.07

Table S7. Results of testing differences between active and inactive compounds in each of the four datasets using the Mann–Whitney U test. The table presents *p*-values. where '_a' denotes active and '_i' denotes inactive.

.	FabI_a vs. FabI_i	FabV_a vs. FabV_i	InhA_a vs. InhA_i	FabK_a vs. FabK_i
pIC50	0	0.022	0	0
SlogP	0.038	0.833	0.291	0.252
TPSA	0.733	0.247	0	0.145
ExactMW	0	0.833	0	0.112
NumRotatableBonds	0.489	0.888	0	0.029
NumHBD	0.357	0.35	0	0.088
NumHBA	0.951	0.339	0.096	0.013
NumAmideBonds	0	1	0	0.213
NumStereocenters	0.28	1	0	1
NumRings	0	1	0	0.811
NumAromaticRings	0.862	1	0	0.584
NumAliphaticRings	0	1	0	0.241
BertzCT	0	1	0	0.761
FCSP3	0.354	1	0	1
fragCpx	0	0.815	0	0.507
nHBDon	0.272	0.35	0	0.914
FilterItLogS	0	0.067	0	0.455
apol	0	0.36	0	0.185
bpol	0	0.805	0	0.419
Radius	0	0.801	0	0.154
ALogP	0	0.197	0	0
AMR	0.424	0.566	0	0.003
FMF	0	0.11	0	0
nAtomLC	0	0.486	0	0.009
nAtomP	0.514	1	0.043	0.42
nAtomLAC	0	0.017	0	0.001
RotBFrac	0	0.04	0	0.058
RotBtFrac	0	0.04	0	0.132
topoShape	0.397	1	0	0.859
AMW	0	0.365	0	0.001

Temporal Progression of Inhibitors' Physicochemical Features for Each Enzyme

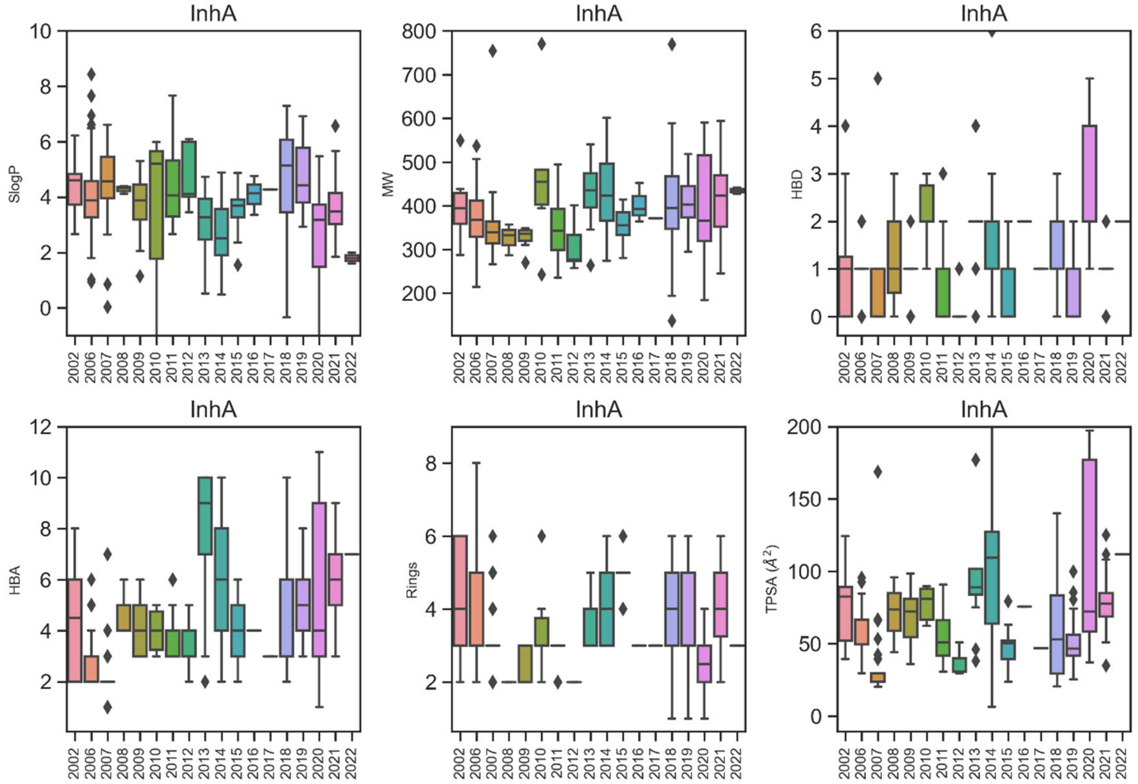

Figure S24. Temporal changes in physicochemical properties of InhA inhibitors.

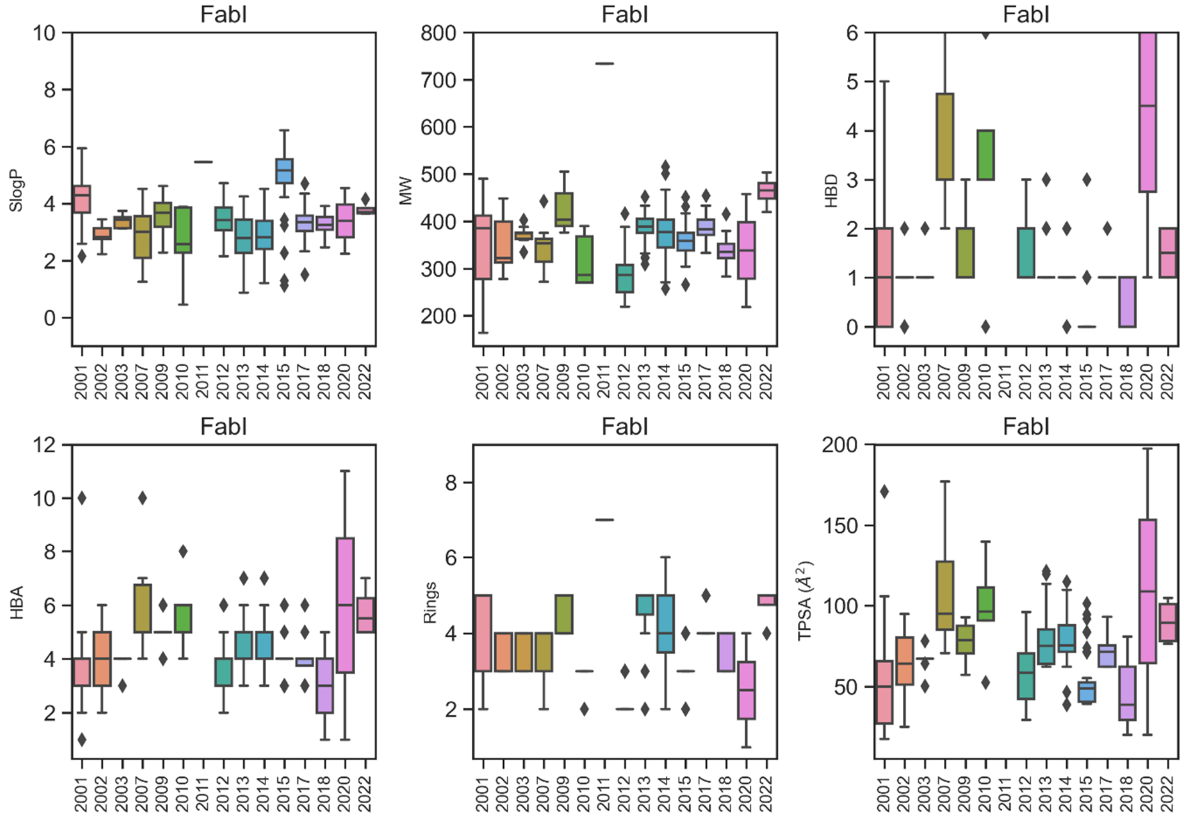

Figure S25. Temporal changes in physicochemical properties of FabI inhibitors

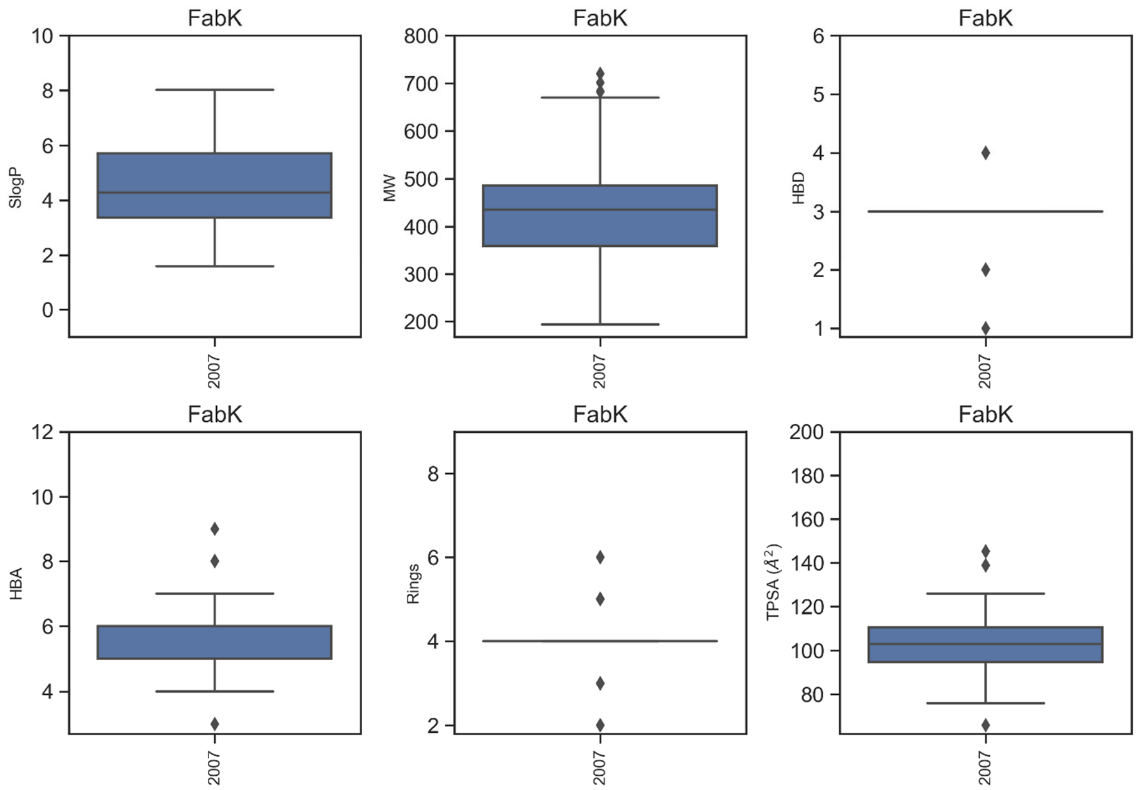

Figure S26. Temporal changes in physicochemical properties of FabK inhibitors.

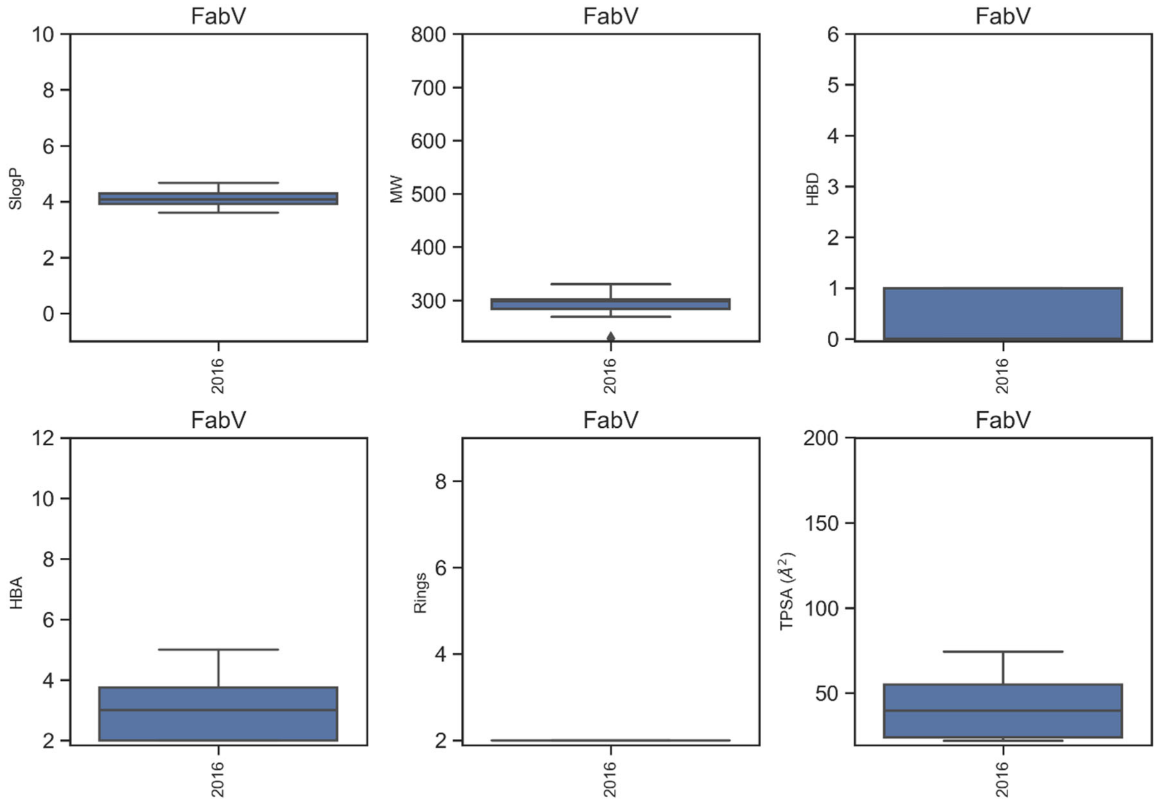

Figure S27. Temporal changes in physicochemical properties of FabV inhibitors.

Lipinski violations

Table S8. Statistics on Lipinski violations.

Enzyme	Activity	Lipinski violations	Total number of compounds	Percent [%]
FabI	Active	0	224	15.9
	Active	1	42	3
	Inactive	0	128	9.1
	Inactive	1	21	1.5
	Inactive	3	1	0.1
FabK	Active	0	20	1.4
	Active	2	3	0.2
	Active	3	1	0.1
	Inactive	0	11	0.8
	Inactive	2	5	0.4
FabV	Active	0	1	0.1
	Inactive	1	2	0.1
InhA	Active	0	164	11.6
	Active	1	92	6.5
	Active	2	6	0.4
	Inactive	3	1	0.1
	Inactive	4	1	0.1
	Inactive	0	475	33.6

Pan-assay interference compounds (PAINS) and Brenk compounds

Table S9. Statistics on compounds classified as PAINS.

Enzyme	Activity	PAINS	Total number of compounds	Percent [%]
FabI	Active	No	236	16.7
	Active	Yes	30	2.1
	Inactive	No	132	9.3
	Inactive	Yes	18	1.3
FabK	Active	No	24	1.7
	Inactive	No	16	1.1
FabV	Active	No	2	0.1
	Active	Yes	1	0.1
	Inactive	No	6	0.4
	Inactive	Yes	1	0.1
InhA	Active	No	258	18.3
	Active	Yes	6	0.4
	Inactive	No	613	43.4
	Inactive	Yes	69	4.9

Table S10. Statistics of compounds classified as Brenk.

Enzyme	Activity	Brenk	Total number of compounds	Percent [%]
FabI	Active	No	91	6.4
	Active	Yes	175	12.4
	Inactive	No	74	5.2
	Inactive	Yes	76	5.4
FabK	Active	No	22	1.6
	Active	Yes	2	0.1
	Inactive	No	15	1.1
	Inactive	Yes	1	0.1
FabV	Active	No	1	0.1
	Active	Yes	2	0.1
	Inactive	Yes	7	0.5
InhA	Active	No	186	13.2
	Active	Yes	78	5.5
	Inactive	No	375	26.6
	Inactive	Yes	307	21.7

Table S11. Combined table for compounds were classified as PAINS and Brenk at once. The total number of compounds left alfer applying both filters is 746.

Enzyme	Activity	PAINS_Brenk	Total number of compounds	Percent
FabI	Active	No	68	4.8
FabI	Active	Yes	198	14
FabI	Inactive	No	66	4.7
FabI	Inactive	Yes	84	5.9
FabK	Active	No	22	1.6
FabK	Active	Yes	2	0.1
FabK	Inactive	No	15	1.1
FabK	Inactive	Yes	1	0.1
FabV	Active	No	1	0.1
FabV	Active	Yes	2	0.1
FabV	Inactive	Yes	7	0.5
InhA	Active	No	186	13.2
InhA	Active	Yes	78	5.5
InhA	Inactive	No	362	25.6
InhA	Inactive	Yes	320	22.7

Table S12. The most common unwanted groups found in ENR inhibitors.

PAINS		Brenk			
**PAINS description**	**No.**	**Brenk description**	**No.**	**Brenk description**	**No.**
ene_rhod_A	84	Michael_acceptor	265	iodine	3
indol_yl_alk	74	Oxygen-nitrogen_single_bond	123	triple_bond	3
dyesA	20	Aliphatic_long_chain	118	Michael_acceptor_	2
catechol_A	16	nitro_group	79	acyl_hydrazine	2
ene_five_het_B	16	Thiocarbonyl_group	55	diketo_group	2
anil_no_alk	14	imine	48	hydrazine	2
anil_di_alk_A	8	beta-keto/anhydride	34	Azido_group	1
anil_di_alk_B	6	phthalimide	24	Carbo_cation/anion	1
mannich_A	6	-halo_pyridine	19	Sulfonic_acid_	1
anil_di_alk_D	2	aniline	19	acyclic_C=C-O	1
anil_di_alk_I	2	isolated_alkene	18	aldehyde	1
azo_A	2	Polycyclic_aromatic_hydrocarbon	15	diazo_group	1
hzone_phenol_A	2	heavy_metal	12	halogenated_ring_	1
hzone_pipzn	2	hydroquinone	11	het-C-het_not_in_ring	1
keto_naphthol_A	2	catechol	9	hydroxamic_acid	1
pyrrole_C	2	cumarine	6	phenyl_carbonate	1
thiophene_amino_C	2	phosphor	6	polyene	1
		phenol_ester	5	quaternary_nitrogen_	1
		oxime_	4	stilbene	1
		quaternary_nitrogen_	4	thioester	1

Molecular similarity

Table S13. Pairwise intra-library Tanimoto similarity values for four analyzed enzyme databases.

	MACCS fingerprints					ECFP4 fingerprints				
**Enzyme**	**Median**	**Max**	**Min**	**Q1**	**Q3**	**Median**	**Max**	**Min**	**Q1**	**Q3**
InhA	0.45	1	0.11	0.32	0.56	0.13	1	0.04	0.1	0.17
FabV	0.74	0.74	0.35	0.4	0.59	0.72	0.72	0.25	0.26	0.49
FabK	0.70	1	0.41	0.58	0.81	0.42	0.88	0.16	0.3	0.6
FabI	0.56	1	0.09	0.37	0.69	0.16	1	0.04	0.12	0.35

Max maximum. Min minimum. Q quartile

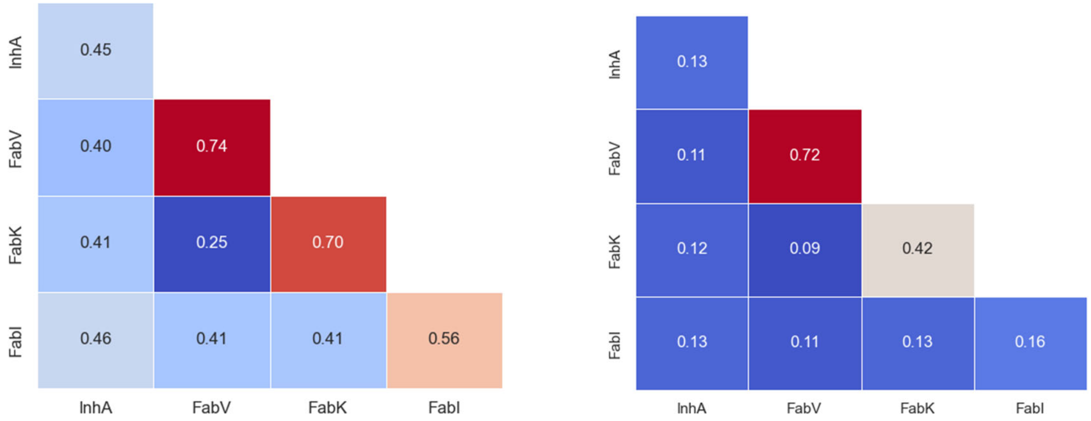

Figure S28. Intra- and inter-group similarity. The diagonal in matrix depicts intra-library comparisons. i.e., the similarity between the compounds in each enzyme. Dark red scores indicate larger similarity while dark blue colors indicate smaller similarity. Tanimoto similarity based on (A) MACCS keys (166-bit) fingerprints and (B) ECFP4 keys fingerprints.

Molecular complexity

Table S14. Correlation between pIC_{50} and BertzCT in actives and inactives of each dataset.

Enzyme	Activity	Correlation	*p*-value	Median
FabI	Active	0.104	0.09	977.0
FabI	Inactive	0.139	0.09	861.5
FabK	Active	0.255	0.23	1072.0
FabK	Inactive	0.211	0.43	1090.0
FabV	Active	0.189	0.88	660.0
FabV	Inactive	0.259	0.58	627.0
InhA	Active	0.340	0.00	1004.0
InhA	Inactive	−0.107	0.01	868.5

Analysis of clusters

Table S15. Analysis of physicochemical properties in the five largest clusters for InhA, FabI, FabV and FabK, including NRB, HBD and HBA.

Enzyme	Cluster	NRB	HBD	HBA	NumRings	NumAromaticRings	SlogP	MW	Lipinski
FabI	1	3.8	1.2	4.4	4.4	2.2	4.1	460.2	0
FabI	2	4.6	1.3	3.7	2.4	2.3	4	250.1	0.2
FabI	3	4.8	0.1	3.9	3.1	3	5.1	375	0.6
FabI	4	3.2	1.6	3.8	4.8	3.8	4.3	398.2	0.5
FabI	5	3.4	0.2	3.2	3.1	3.1	4	254.1	0.2
FabK	1	6	2.9	5.9	4.2	4.2	5.2	486	0.4
FabK	2	4	1.3	4	4	3	3.1	374.2	0
FabV	1	7.2	0.5	3	2	2	3.9	300.2	1
InhA	1	3.6	0.9	2.6	4.2	2	5.8	454.2	0.2
InhA	2	6.2	0.9	3.5	2.5	2.5	6.1	348.1	0.6
InhA	3	5.3	1.2	4	3.3	3	5.1	486.2	0.3
InhA	4	7.2	2.1	7.4	4.8	3.6	1.3	555.2	0.5
InhA	5	3.8	0.5	2.5	3.9	2.9	5	431.2	0.2

Matched molecular pairs (MMP)

Our focus has been on the identification of activity cliffs formed by similar compounds with notable differences in biological activity [1]. To systematically extract matched molecule pairs (MMPs) in four ENR datasets, we implemented and modified the algorithm of Hussain and Rea [2]. During the fragmentation process, we have allowed a molecule to fragment at a different number of single bonds, which is referred to as the number of cuts. The result was the generation of a total of 96,264 distinct pairs, most of which have a nearly symmetric distribution of ΔpIC50 with the center at 0 (Figure S29). We identified 2314 unique significant transformations in all four datasets combined, namely 728, 1612, 8, and 4 for FabI, InhA, FabK, and FabV, respectively (Supplementary Materials, Table S16). It is important to note that the number of compounds forming similarity pairs is actually smaller than the number of pairs, as individual molecules can be part of multiple pairs. Thus, we were able to identify 950 compounds involved in at least one transformation with a significant effect, which corresponds to 63 % of all compounds. Although we analyzed all four different enzyme datasets, we actually found MMPA less useful for smaller datasets such as FabK and FabV. However, due to the limited information obtained from previous analyzes, we still analyzed the MMPs of FabK and FabV.

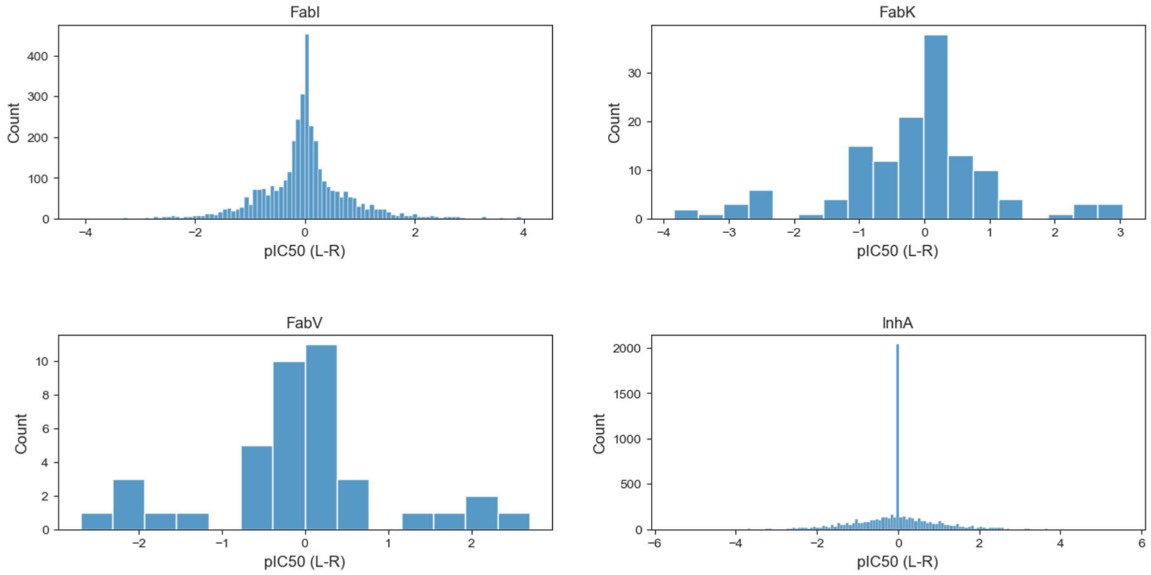

Figure S29. Histogram of matched molecular pairs: distribution of significant and non-significant transformations.

Table S16. Number of pairs identified by MMP analysis

Enzyme	Total number of unique pairs (S+NS)	No of unique pairs
FabI	32322	728
InhA	63582	1612
FabK	2056	8
FabV	162	4

Both Figure S30 and Figure S31 illustrate the type of transformation and indicate whether it is a terminal transformation (number of cuts = 1) or a core transformation (number of cuts > 1). The data show that core transformations are more common in FabI, while terminal transformations are more common in InhA, although both types are adequately represented in each data set. The maximum number of pairs found for InhA, FabI, FabK and FabV was 200, 60, 10 and 15, respectively, which, as expected, corresponds well with the size of each dataset (Figure S30). In contrast to previous reports [3], where there were numerous examples for only a few transformations, the current dataset has a larger number, with a median of 10 to 15 pairs per transformation.

It is important to acknowledge that the analysis provided only limited information due to the limited scope of the FabK and FabV datasets. Only core transformations were observed in the FabK dataset, and only end transformations were observed in the FabV dataset. This observation can be attributed to the limited availability of meaningful structure-activity relationship (SAR) data.

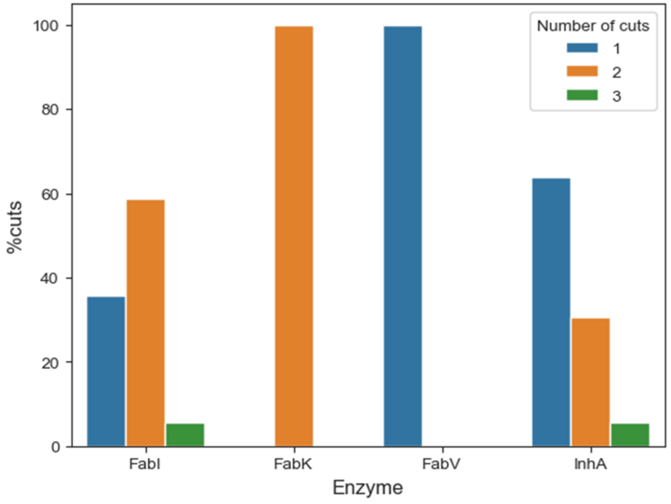

Figure S30. Distribution of cut types across enzyme datasets.

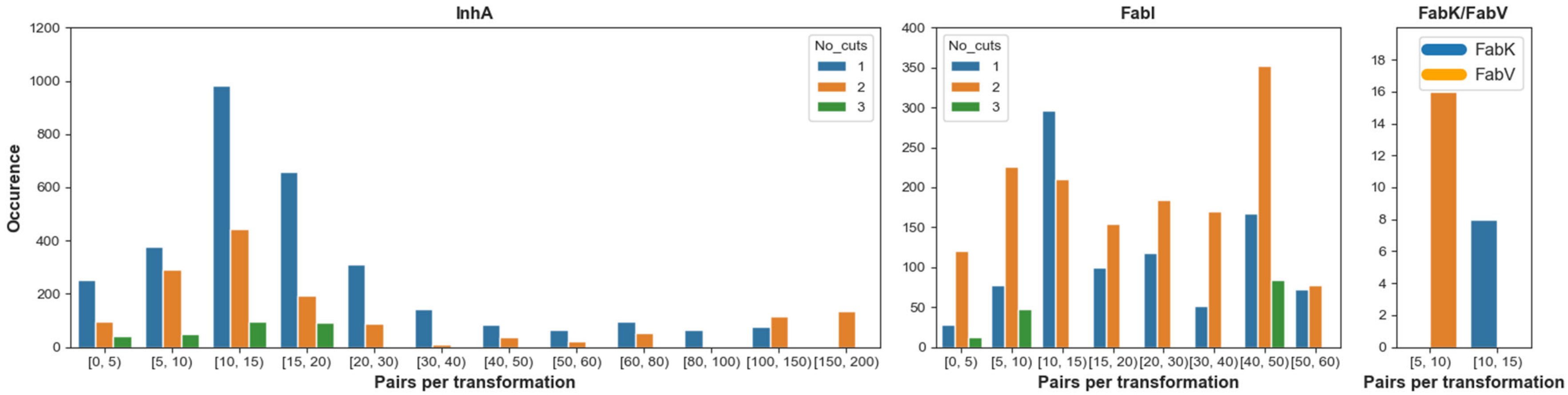

Figure S31. Distribution of the number of pairs per transformation with significant effect across all enzyme datasets.

Table S17. Most frequent MMP transformations.

Transformation	ΔpIC_{50}	SD	No.	Transformation	ΔpIC_{50}	SD	No.
**FabI**				**InhA**			
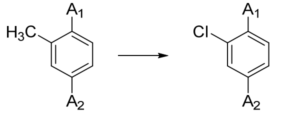	0.116	0.252	59	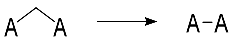	0.194	0.850	187
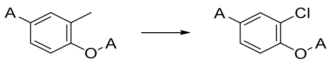	0.135	0.261	54	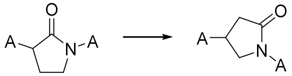	0.290	0.566	110
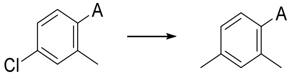	0.219	0.338	54	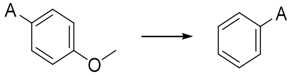	0.322	0.863	110
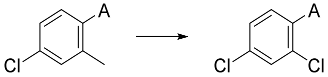	0.135	0.261	54	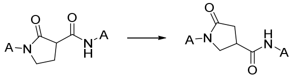	0.392	0.606	102
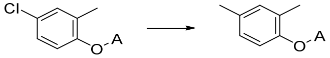	0.219	0.338	54	A−Br → A−Cl	0.181	0.351	102
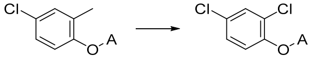	0.135	0.261	54	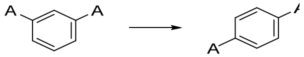	0.180	0.576	100
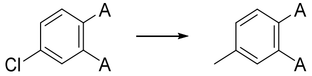	0.278	0.369	51	A−Cl → A−F	0.127	0.467	96
A−A → 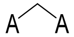	0.187	0.895	48	A−Cl → A−F	0.082	0.473	80
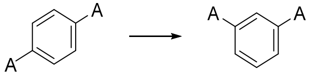	0.659	0.812	48	$A-CH_3$ → A−F	0.082	0.473	80
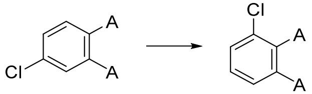	0.470	0.367	42	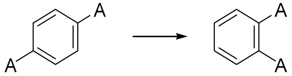	0.265	0.478	74
**FabV**				**FabK**			
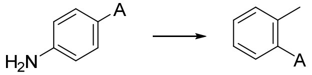	1.263	1.403	11	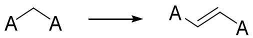	1.464	1.454	7
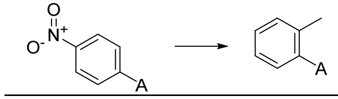	1.190	1.081	11	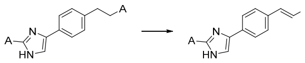	1.575	2.036	7

Table S18. Transformations with highest proportion of activity cliffs. SD= Standard deviation.

Transformation	ΔpIC_{50}	SD	No.	%Activity cliffs
**FabI**				
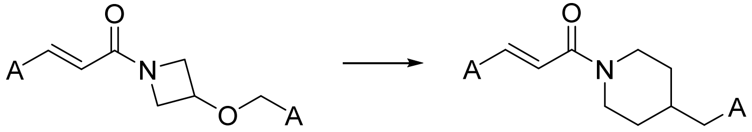	1.610	0.555	10	10
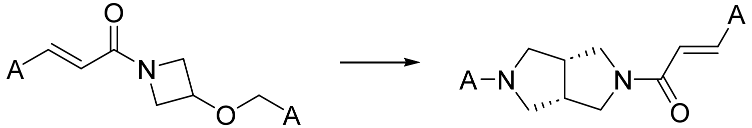	1.868	0.376	10	10
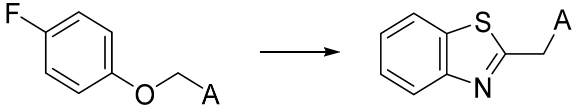	1.521	1.385	10	10
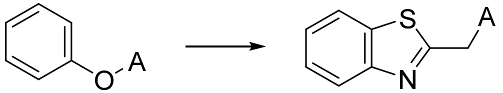	1.104	1.267	10	10
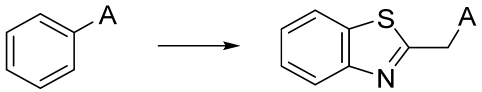	1.503	1.143	10	10
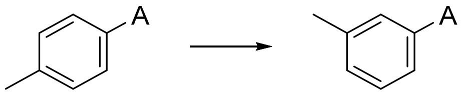	1.633	0.570	11	9.1
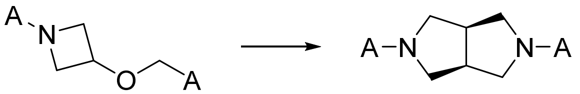	1.715	0.376	12	8.3
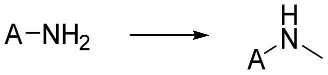	1.334	1.334	14	7.1
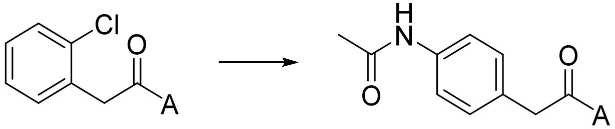	1.602	0.699	15	6.7
**InhA**				

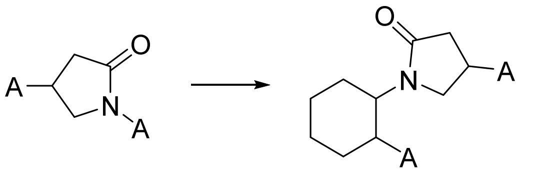	2.642	0.543	15	20
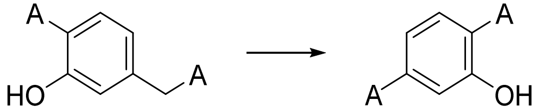	2.265	0.128	15	20
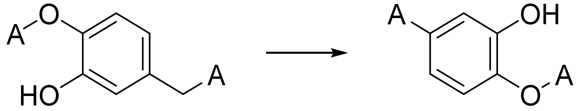	2.265	0.128	15	20
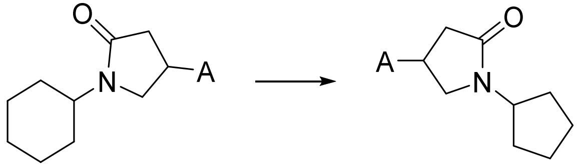	2.642	0.543	15	20
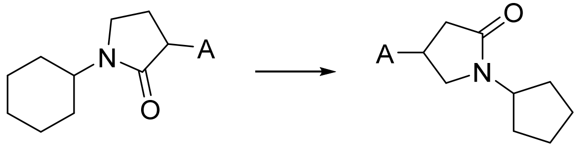	2.463	0.367	15	20
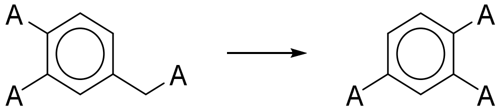	2.265	0.128	15	20
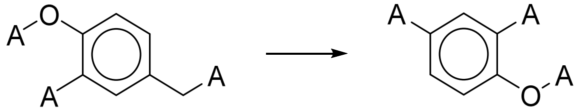	2.265	0.128	15	20
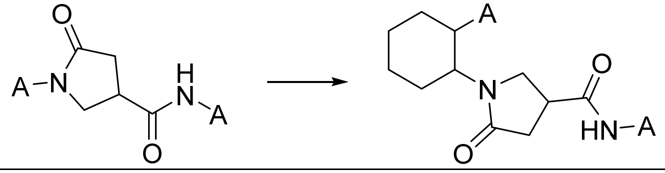	2.901	0.434	10	20

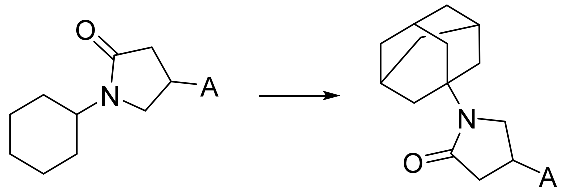	2.666	0.766	10	20
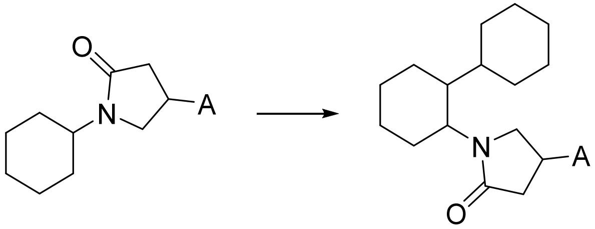	2.666	0.766	10	20
**FabV**				
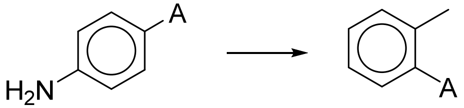	1.263	1.403	11	9.1

Table S19. Physicochemical properties of fragments in activity cliffs.

Enzyme	SlogP	TPSA	MW	NRB	NumHBD	NumHBA	NumAmideBonds	FractionCSP3
FabI	1.829	17.07	148.1	1	0	1	0	0.333
FabK	2.377	14.34	122.1	1.5	0.5	0.5	0	0.404
FabV	1.903	26.02	107.1	0	0	1	0	0.143
InhA	2.004	18.14	152.1	1	0	1	0	0.333

NRB = Number of rotatable bonds

SHAP analysis

Table S20. Metrics for classification models and SHAP analysis with special consideration of Random Forest (RF).

Classifier	Train_test	Feature selection	Outlie removal technique	ROC AUC	MCCC	F1	F-beta	Precision	Recall	Accuracy	Num features
gbc	test	rfe	LocalOutlierFactor	0.981	0.765	0.709	0.668	0.647	0.831	0.868	25
gbc	train	rfe	LocalOutlierFactor	0.997	0.968	0.96	0.974	0.974	0.974	0.974	25
rf	test	selmodel	None	0.979	0.789	0.769	0.745	0.732	0.831	0.885	76
rf	train	selmodel	None	0.996	0.961	0.956	0.968	0.969	0.967	0.968	76
xgb	test	rfe	LocalOutlierFactor	0.976	0.76	0.683	0.643	0.623	0.827	0.861	25
xgb	train	rfe	LocalOutlierFactor	0.996	0.961	0.952	0.968	0.969	0.968	0.968	25
knn	test	rfe	LocalOutlierFactor	0.957	0.698	0.595	0.565	0.552	0.800	0.807	25
knn	train	rfe	LocalOutlierFactor	0.990	0.951	0.944	0.960	0.961	0.959	0.960	25
lgb	test	rfe	LocalOutlierFactor	0.968	0.723	0.669	0.625	0.605	0.883	0.832	25
lgb	train	rfe	LocalOutlierFactor	0.993	0.941	0.942	0.951	0.953	0.951	0.952	25
et	test	selmodel	Dummy	0.971	0.734	0.760	0.704	0.677	0.923	0.843	76
et	train	selmodel	Dummy	0.993	0.941	0.937	0.951	0.953	0.95	0.952	76
dt	test	selmodel	Dummy	0.941	0.714	0.691	0.661	0.646	0.794	0.827	76
dt	train	selmodel	Dummy	0.994	0.958	0.934	0.965	0.966	0.965	0.966	76
ada	test	rfe	LocalOutlierFactor	0.778	0.242	0.408	0.409	0.417	0.489	0.567	25
ada	train	rfe	LocalOutlierFactor	0.888	0.657	0.689	0.719	0.769	0.699	0.710	25

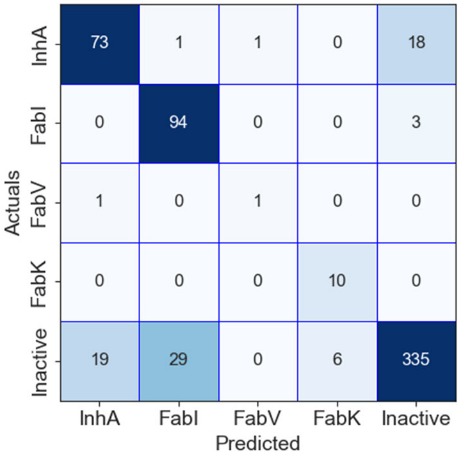

Figure S32. Confusion matrix for random forest classifier.

References:

1. Yang, Z.; Shi, S.; Fu, L.; Lu, A.; Hou, T.; Cao, D. Matched Molecular Pair Analysis in Drug Discovery: Methods and Recent Applications. *J. Med. Chem.* **2023**, *66*, 4361–4377. https://doi.org/10.1021/acs.jmedchem.2c01787.
2. Hussain, J.; Rea, C. Computationally Efficient Algorithm to Identify Matched Molecular Pairs (MMPs) in Large Datasets. *J. Chem. Inf. Model.* **2010**, *50*, 339–348. https://doi.org/10.1021/ci900450m.
3. Sullivan, T.; am Ende, C.; Truglio, J.; Johnson, F.; Lenaerts, A.; Slayden, R.; Kisker, C.; Tonge, P. High Affinity InhA Inhibitors with Activity against Drug-Resistant Strains of Mycobacterium Tuberculosis. *ACS Chem Biol*. **2006**, *1*, 43-53. doi: 10.1021/cb0500042.
